# Hormonal contraceptive use is associated with differences in women’s inflammatory and psychological reactivity to an acute social stressor

**DOI:** 10.1016/j.bbi.2023.10.033

**Published:** 2023-10-31

**Authors:** Summer Mengelkoch, Jeffrey Gassen, George M. Slavich, Sarah E. Hill

**Affiliations:** a Department of Psychiatry and Biobehavioral Sciences, University of California, Los Angeles, 760 Westwood Plaza, Los Angeles, CA 90095, United States; b Department of Psychology, Texas Christian University, 2955 South University Drive, Fort Worth TX 76129, United States

**Keywords:** Stress, Hormonal Contraceptives, Inflammation, Glucocorticoid, Women’s Health

## Abstract

Women using hormonal contraceptives (HCs) exhibit numerous signs of chronic inflammation, including elevated C-reactive protein levels and greater risk of developing mood and autoimmune disorders. However, users and non-users of HCs often have similar circulating proinflammatory cytokine levels, making the mechanism of association unclear. One possible explanation for this paradox is that HC users exhibit differences in their inflammatory responses to psychosocial stress that, over time, could contribute to chronic inflammation and its pathologies. Here, we tested this possibility by examining women’s glucocorticoid, inflammatory, and psychological responses to the Trier Social Stress Test (TSST) in 67 naturally cycling (NC) and 60 oral HC-using women (*M*_*age*_ = 19.31, *SD*_*age*_ = 1.95). As hypothesized, HC users and NC women exhibited different glucocorticoid and proinflammatory cytokine responses to the TSST. For NC women, TSST-induced increases in glucocorticoids were uncommon, and increases in glucocorticoids were accompanied by elevations in IL-6. In contrast, for women using HCs, increases in glucocorticoids in response to the TSST were common, and increases in glucocorticoids were accompanied by increases in TNF-α. HC users and NC women also differed in their psychological responses to the TSST, with HC users reporting elevated stress levels compared to NC women. Together, these results suggest that HC use impacts women’s glucocorticoid, inflammatory, and psychological responses to psychosocial stress, potentially contributing to observed differences in these women’s mental and physical health.

## 1. Introduction

More than 80% of women in the United States report having used hormonal contraceptives (HCs) at some point during their reproductive-aged years (Daniels & Jones, 2013), and there are presently more than 300 million HC users worldwide (United Nations, 2019). Despite being widely used and extensively tested for safety and efficacy, little research has been dedicated to understanding the non-life-threatening physiological and behavioral consequences of HC use (for discussions, see Hill and Mengelkoch, 2023; Montoya & Bos, 2017; Pletzer & Kerschbaum, 2014; Rrapaj et al., 2023). Such work is necessary, however, as a small, but growing body of research suggests that HC use may have multiple unintended physiological and psychological effects on women, including those related to inflammation and stress reactivity (e.g., Kirschbaum et al., 1999; Larsen et al., 2020; Lovallo et al., 2019; Masama et al., 2022; Nielsen et al., 2013; Roche et al., 2013). In the present study, we build on this research, investigating the association between HC use and women’s physiological and psychological responses to an acute social stressor. In doing so, we sought to identify a potential mechanism that may contribute to the often-observed associations between HC use and elevated risk of inflammation-related health outcomes (e.g., Costenbader et al., 2007; Khalili et al., 2013; Okoth et al., 2020; Skovlund et al., 2016).

### 1.1. Hormonal Contraceptive Use, Inflammation, and Health

HCs suppress women’s endogenous sex steroid hormone production, replacing these hormones with synthetics. Sex steroid hormones are functionally pleiotropic, and have a wide range of effects on multiple bodily systems, including the immune system and inflammatory activity (Klein & Flanagan, 2016). For example, researchers have found that endogenous estrogen levels are negatively associated with levels of C-reactive protein (CRP; Gaskins et al., 2012), a marker of chronic inflammation. However, researchers have found the opposite association between exogenous hormone administration and levels of CRP (Pradhan et al., 2002; Williams et al., 2016), suggesting that the impact of sex steroid hormones on bodily inflammatory activity may depend, alongside other factors, on their source.

These inflammatory outcomes of exogenous hormone administration may lead to long-term mental and physical health risks in those using HCs, as chronic systemic inflammation takes a toll on the body (Slavich, 2015). Despite being the immune system’s first line of defense against injury and infection, over time, elevated inflammatory activity increases a person’s risk of developing autoimmune disorders (Duan et al., 2019) and diseases of aging, including coronary heart disease, cancer, and neurodegenerative disease (Furman et al., 2019). Elevated inflammation is also regularly observed in those with mood disorders (Capuron & Miller, 2011; Dantzer et al., 2008; Dooley et al., 2018; Miller & Raison, 2016; Raison & Miller, 2011; Slavich & Irwin, 2014), highlighting the role that inflammation plays in both physical and mental health.

Recently, researchers have begun to explore the possibility that HC use may increase women’s risk of chronic inflammation. Consistent with this view, research finds that HC users exhibit higher levels of each oxidative stress (Cauci et al., 2021) and CRP (Larsen et al., 2020; Masama et al., 2022) compared to what is observed in naturally cycling (NC) women. Others find that women using HCs are at an elevated risk of developing inflammation-related disorders, including cardiovascular disease (e.g., Jamil & Siddiq, 2012; Okoth et al., 2020), depression (e.g., Bengtsdotter et al., 2018; Masama et al., 2022; Skovlund et al., 2016), and autoimmune disease (e.g., systemic lupus erythematosus: Costenbader et al., 2007; Chron’s disease: Khalili et al., 2013). Indeed, doctors often recommend that women with certain autoimmune disorders do not use HCs, as they can worsen symptoms (e.g., Benagiano et al., 2019).

Despite being linked to elevated levels of CRP (Divani et al., 2015; Larsen et al., 2020; Masama et al., 2022) and inflammation-mediated health risks, HC use does not consistently predict basal (i.e., unstimulated) levels of the key inflammatory cytokines interleukin-1β (IL-1β), interleukin-6 (IL-6), or tumor necrosis factor-α (TNF-α), which have been found to be roughly the same between HC users and non-users (Caputi et al., 2022; Eagan et al., 2021; Larsen et al., 2020). Although the exact reason for this paradox remains unclear, one potential explanation is that HC users may have similar basal inflammation levels as non-users but exhibit exaggerated inflammatory responses to immune-activating challenges, including acute psychosocial stressors, which have been shown to strongly upregulate inflammatory activity (e.g., Slavich et al., 2010).

### 1.2. Acute Stress Reactivity

A sizeable body of research has shown that psychosocial stressors are a potent activator of both the hypothalamic–pituitary–adrenal (HPA) axis and the immune system (Marsland et al., 2017; Slavish et al., 2015; Steptoe et al., 2007). Although research examining links between HC use and women’s inflammatory response to stress is lacking, many studies have found differences in HPA axis functioning between HC users and NC women. For example, research has found that HC users exhibit a blunted glucocorticoid (GC) response to acute stressors relative to both men (Kirschbaum et al., 1999; Lovallo et al., 2019; Nielsen et al., 2013; Roche et al., 2013) and NC women after exposure to an acute social stressor (Kirschbaum et al., 1999; Lovallo et al., 2019; Roche et al., 2013), the socially evaluated cold-pressor test (Merz, 2017), the cold-pressor stress task (Nielsen et al., 2013), and administration of naltrexone, a drug that increases cortisol levels (Roche et al., 2013). Blunted GC reactivity to stressors is associated with health issues, including chronically heightened inflammation (e.g., Miller et al., 2008) and autoimmune disorders (e.g., atopic dermatitis; Buske-Kirschbaum et al., 1997). Typically, GC and inflammatory responses to acute stress are inversely related (Slavich & Irwin, 2014), with those displaying a blunted GC response to stress also exhibiting an exaggerated inflammatory response to stress (Kunz-Ebrecht et al., 2003). Accordingly, in addition to a dysregulated GC response, women using HCs may exhibit a dysregulated inflammatory response to acute psychosocial stressors.

No studies have examined inflammatory responses to an acute social stressor in HC users *in vivo*. However, one study has found that women using HCs have different patterns of stimulated IL-6 production after stress *ex vivo* (i.e., in whole blood) compared to NC women (Rohleder et al., 2003). Although these differences were not statistically significant, they nevertheless provide preliminary evidence that HC use may – in addition to predicting a dysregulated HPA axis response to stress – predict dysregulation in women’s inflammatory response to acute stress.

### 1.3. The Present Study

The goal of the present study was to replicate past research examining associations between HC use and GC responses to stressors (e.g., Kirschbaum et al., 1999; Lovallo et al., 2019; Nielsen et al., 2013; Roche et al., 2013), and to extend this work by examining whether differences in HC users’ and NC women’s GC responses to stress impact their inflammatory response to acute psychosocial stress. Guided by insights from prior research, we hypothesized that HC users would exhibit a blunted GC and exaggerated inflammatory response to an acute psychosocial stressor relative to NC women. Further, we hypothesized that these differences would correspond to differences in women’s subjective responses to the stressor, with HC users exhibiting more negative mood and subjective stress following the stressor relative to what is observed in NC women.

## 2. Method

### 2.1. Participants

Participants were 153 women recruited from a private university in the southern United States and surrounding community between August 2021 and April 2022. All NC women (*n* = 75) reported that they had not used HCs within the last year. All women using HCs were current users of first, second, or third generation combined oral contraceptive pills (*n* = 78) who had been on them for at least three months. We sought to maximize sample size given time and monetary constraints. All NC women were scheduled to participate in the luteal phase of the ovulatory cycle, between days 20 and 24, as determined via a forward counting method. This cycle phase was chosen because past research indicates that the largest differences between the stress responses of HC and NC women occur during the luteal (compared to follicular) phase of the ovulatory cycle (e.g., Kirschbaum et al., 1999). HC users were scheduled at the same time in their “cycle” as NC women and came into the laboratory between days 20 and 24 of their pill-pack, reflecting an active pill phase of their HC treatment.

Before enrolling in the study, women were prescreened and asked the start date of their most recent menstrual cycle, regularity of their cycle, and length of their typical ovulatory cycle. Women were excluded if they: (1) took medications known to impact inflammation or the stress response, (2) had a chronic medical condition, including any endocrine disorders, (3) self-reported a body mass index (BMI) greater than 30^1^, (4) had an acute illness, (5) were pregnant or breastfeeding, or (6) did not adhere to pre-study procedures (i.e., did not fast, did not abstain from alcohol, anti-inflammatory medications, and vigorous exercise for at least 12 h before the session). All participants were compensated with course credit or a gift card. Following participation in the study, participants were excluded from data analysis if, during their session, they reported being on a fourth generation HC (*n* = 3) or a medication that would have disqualified them from participation (*n* = 23), including anxiety medications/beta-blockers (*n* = 4), stimulants (*n* = 8), oral acne medication (*n* = 6), and antipsychotics (*n* = 5). The final data analytic sample was comprised of 127 women (HC: *n* = 60; NC: *n* = 67). See Table 1 for characteristics of the sample.

### 2.2. Procedure

All study sessions began between 7 and 9am to minimize the impact of circadian rhythms on measured outcomes. When participants arrived for their sessions, researchers first took steps to ensure that participants were free from coronavirus symptoms and fever (i.e., had a temperature below 99.9 degrees Fahrenheit) and had complied with pre-study procedures. Symptomatic and noncompliant participants were rescheduled and dismissed.

Once the study session began, participants provided informed consent, completed a brief baseline survey (including measures of current mood and stress levels rated using a visual analogue scale), and had their height, weight, and blood glucose levels measured. Blood glucose measurements were obtained using a standard finger prick using commercially available glucose tests strips and glucometers. Participants with a blood glucose level above 105^2^ were considered pre-diabetic and were thanked and dismissed (or rescheduled if they indicated that they had not fasted).

Next, participants provided a 4 mL passive drool saliva sample (baseline) before being escorted to a separate room to complete the Trier Social Stress Test (TSST; Kirshbaum et al., 1993; described below). Following the TSST, participants completed measures assessing their levels of mood and stress. Participants then completed a short battery of questions measuring other constructs unrelated to the present research study. Fifteen minutes after completing the TSST, participants provided a second 4 mL saliva sample (post-stress) and again reported on their levels of mood and stress. Finally, participants completed questionnaires about their history of HC use and demographic information before providing a final assessment of their mood and stress levels. At the end of the study, participants were orally debriefed, thanked, and compensated.

### 2.3. Measures

#### 2.3.1. Baseline Measures

Prior to the TSST, participants completed baseline measures to assess their recent behaviors, health history, current mood, and stress levels. Specifically, participants were asked to list any chronic illnesses, current medication use, medication they had used within the last 12 h, recent illnesses (within last week, and last illness), and recent vaccination history (within last two weeks, and date of last coronavirus vaccine). They were also asked to report the number of hours they slept the night before, how long it had been since they had eaten, and the number of alcoholic beverages they had consumed and number of hours of exercise they had engaged in during the last 48 h.

#### 2.3.2. Visual Analogue Scales: Subjective Positivity of Mood and Subjective Stress Measures

Prior to the TSST, immediately following the TSST, 15 min after the TSST, and approximately 50 min after the TSST (at the end of the study session), participants indicated their current mood and stress levels on a visual analogue scale by responding to the questions, “How would you rate your mood right now?” (endpoints: *Very negative*, *Very positive*), and “How stressed do you feel right now?” (endpoints: *Not at all stressed*, *Extremely stressed*). Participants were instructed to mark a vertical line indicating their current mood and stress levels on a horizontal line ranging from the endpoints listed above for each question. These marks were then measured in centimetres, providing a continuous measure of mood and stress levels between 0 and 11 cm at four timepoints throughout the study session.

#### 2.3.3. Trier Social Stress Test

To induce stress, participants were brought into a conference room where they were seated and instructed to prepare a five-minute speech about why they deserve their dream job, which would be recorded and sent to an independent review team for evaluation. Participants then delivered their speech in front of a HC status-blind researcher who was trained to refrain from providing positive affirmation while the participant was supposedly being recorded by the video camera that was positioned on a tripod next to the researcher. If a participant stopped speaking before five minutes had passed, the researcher advised the participant that they had time remaining and that they must continue speaking. After the five minutes had elapsed, participants then completed a surprise mental arthritic task, where they were asked to count down from 1022 by 13’s for five minutes. If the participant made a mistake, the researcher informed them that their response was incorrect, and that they needed to start over. Following the TSST, participants completed survey and demographic measures.

#### 2.3.4. Biological Measures

Saliva samples were collected at baseline, prior to introduction of the TSST, and again 15 min after offset of the TSST. Following collection, saliva samples were immediately centrifuged and supernatant was stored at −80 degrees Celsius until later thawed, centrifuged, and assayed in duplicate for the analytes described below.

#### 2.3.5. Cortisol

Saliva samples were assayed for levels of circulating cortisol using commercially available enzyme-linked immunosorbent assay (ELISA) kits (Salimetrics, Carlsbad, CA, United States) per manufacturer instructions. Plates were read using an ELISA machine at 450 nm. The intra-assay coefficient of variation (CV) for these assays was 3.56% and the inter-assay CV was 8.90%.

#### 2.3.6. Cytokines

Saliva samples were assayed for levels of key cytokines IL-1β, IL-6, and TNF-α using commercially available multiplexing assay kits (Miso Scale Delivery [MSD], Rockville, MD, United States) per manufacturer instructions^3^. The intra-assay CV for these assays was 3.02% and the inter-assay CV was 4.73%.

#### 2.3.7. Demographic Measures

Participants completed demographic measures assessing age, race, socioeconomic status (SES), smoking status, sexual activity status, and relationship status.

### 2.4. Data Preparation

All data were assessed for normality and outliers prior to analyses. Cortisol levels and inflammatory biomarkers were positively skewed and therefore log transformed, per convention (e.g., Genser et al., 2007; Stoffel et al., 2021). See Supplemental Materials, Table S1 for skewness and kurtosis statistics before and after transformations. Eight outliers remained across measures (≥3 standard deviations [*SD*s] from the mean). These scores were windsorized to approximate a normal distribution, by replacing these values with values +/−3 *SD*s from the appropriate means (Wilcox, 1994). Next, we computed change scores for cortisol and inflammatory biomarkers to investigate associations between changes in these biomarkers in response to stress by subtracting baseline values from the post-stress values for each variable (e.g., Kapuku et al., 2002; Steptoe et al., 2002). Finally, we computed a change score for subjective appraisals of both stress levels and mood, computed using stress and mood responses occurring concurrently with saliva collection.

### 2.5. Data analytic plan

Data were analysed using IBM’s SPSS statistical package version 25 (IBM Corp., 2018). First, we used two mixed-model 4 (Time, measured within-subjects: baseline vs. immediately post-stress vs. post-stress, vs. end of study) × 2 (HC Use, measured between-subjects: NC vs. HC) analyses of variance (ANOVAs) to examine participants’ subjective stress and mood positivity. Simple effects were investigated using Tukey’s Least Significant Difference follow-up tests to probe differences in marginal means. Next, we investigated differences in cortisol levels and inflammatory biomarkers between NC and HC-using women using a series of mixed-model 2 (Time, measured within-subjects: baseline vs. post-stress) × 2 (HC Use, measured between-subjects: NC vs. HC) ANOVAs on cortisol levels and levels of inflammatory biomarkers.

Following these analyses, we used computed change scores to investigate if HC Use (dummy coded: NC = 0 vs. HC = 1) moderated the associations between changes in cortisol and changes in proinflammatory cytokines in response to the TSST using moderated regression analyses. When significant or trending two-way interactions emerged, we probed these interactions with simple slopes tests to test whether changes in cortisol predicted changes in levels of proinflammatory cytokines in response to stress in each NC women and women using HCs. Additionally, the Johnson-Neyman (J-N) technique (Johnson & Neyman, 1936) was used to identify regions of significance, or at which points along the x-axis (i.e., changes in cortisol levels) NC women and women using HCs exhibited differences in their proinflammatory cytokine responses to stress. Because a value of zero change is meaningful for the current analysis, change scores were not mean centered.

Finally, to investigate whether HC Use moderated the associations between changes in cortisol and changes in each subjective stress and positivity of moods following stress, we used computed change scores for subjective appraisals of stress levels and mood in moderated regression analyses. See Supplemental Materials (*Harmonic Mean P-Value Analysis*) for results of a harmonic mean *p*-value analysis, used to account for any increased familywise error rates given the number of analyses conducted. Data are available at: https://osf.io/syu2x/.

## 3. Results

### 3.1. Subjective Reponses to the Acute Social Stressor

**Fig. 1.**
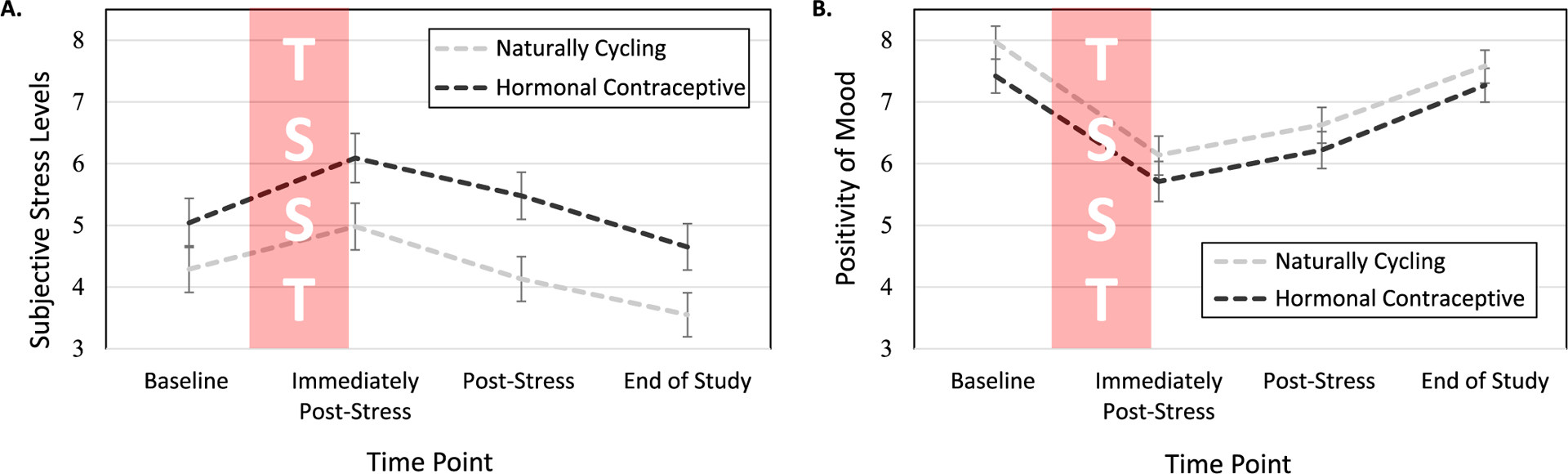
Subjective stress levels and positivity of moods before and after the Trier Social Stress Test (TSST). Women reported increased stress and decreased positivity of moods in response to the TSST. **(A)** HC users reported more TSST-induced stress than did NC women. **(B)** There were no differences in positivity of mood between HC users and NC women. Note. Error bars represent the standard error of the mean; Full scale range: 0–11 cm; NC = naturally cycling; HC = hormonal contraceptive.

See Table 2 and Fig. 1 for descriptive statistics of participants’ subjective responses to the laboratory-based acute social stressor (TSST).

#### 3.1.1. Subjective Stress Levels

A significant main effect of Time on subjective stress levels emerged, *F*(3, 336) = 19.24, *p* ≤ 0.001, $\eta_{\text{p}}^{2}=0.15$. Simple effect analyses revealed that participants reported the highest levels of subjective stress immediately following the TSST (*M* = 5.53, *SE* = 0.28) compared to all other time points, *p*s ≤ 0.002, indicating that the stress manipulation was effective at eliciting stress. Participants reported the lowest levels of subjective stress at the end of the study (*M* = 4.10, *SE* = 0.26) compared to all other time points, *p*s ≤ 0.002. There were no significant differences in subjective stress levels reported at baseline (*M* = 4.66, *SE* = 0.28) compared to post-stress (*M* = 4.81, *SE* = 0.26), *p* =.450.

In addition to the main effect of Time, a significant main effect of HC Use was found for participants’ subjective stress levels, *F*(1, 112) = 4.97, *p* =.028, $\eta_{\text{p}}^{2}=0.04$. Simple effects tests revealed that women using HCs (*M* = 5.31, *SE* = 0.35) reported higher levels of subjective stress throughout the study compared to NC women (*M* = 4.24, *SE* = 0.33). No significant Time × HC Use interaction emerged, *p* =.474.

#### 3.1.2. Subjective Positivity of Mood

As was found for stress, a significant main effect of Time was found for participants’ mood, *F*(3, 339) = 43.13, *p* ≤ 0.001, $\eta_{\text{p}}^{2}=0.28$. Simple effect analyses revealed that participants reported the most negative mood immediately post-stress (*M* = 5.93, *SE* = 0.22) compared to all other time points, *p*s ≤ 0.001. This indicates that the stress manipulation, in addition to successfully increasing participants’ levels of stress, also significantly worsened their moods. Moods reported at baseline (*M* = 7.70, *SE* = 0.19) and at the end of the study (*M* = 7.42, *SE* = 0.19) did not differ significantly from each other, *p* = .148, and were each significantly more positive than what participants reported post-stress (*M* = 6.42, *SE* = 0.21), *p*s ≤ 0.001. Neither a main effect of HC Use nor a significant Time × HC use emerged in predicting women’s subjective mood, *p*s ≥ 0.213. This pattern of results indicates that NC women and women using HCs experienced similar moods throughout the course of the study, despite women using HCs reporting more subjective stress throughout the study compared to NC women.

### 3.2. Biological Responses to the Acute Social Stressor

**Fig. 2.**
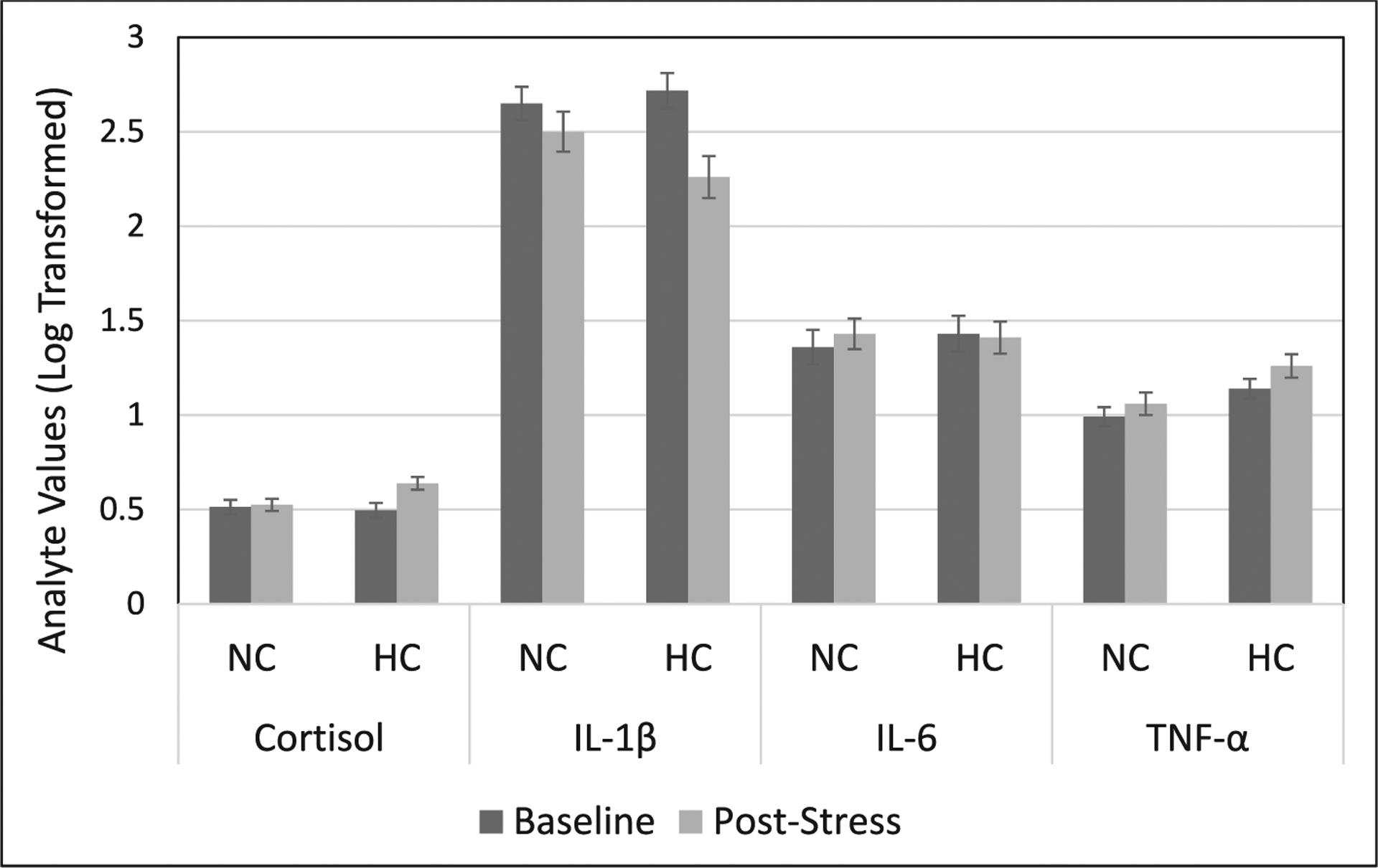
Cortisol and inflammatory responses to the Trier Social Stress Test (TSST), reported with transformed values. HC users exhibited an increase in cortisol and decrease in IL-1β levels following the TSST, in contrast to NC women. HC users also had higher levels of TNF-α both before and after the TSST compared to NC women. Post-Stress assessments taken 15 min after the TSST was completed. Values have been log-transformed and a constant has been added (+1) to all values to improve interpretability of the figure (see Supplemental Materials Table S2 for raw values with appropriate units). Outliers were trimmed to +/−3 standard deviations from the mean. Error bars represent the standard error of the mean. NC = naturally cycling; HC = hormonal contraceptive; IL-1β = interleukin-1β; IL-6 = interleukin-6; TNF-α = tumor necrosis factor-α.

See Table 3 and Fig. 2 for descriptive statistics of biological responses to stress. See Table S2 for raw values (i.e., values prior to transformations) of biological variables.

#### 3.2.1. Cortisol Levels

A significant main effect of Time emerged, *F*(1, 123) = 11.72, *p* =.001, $\eta_{\text{p}}^{2}=0.09$, revealing that cortisol levels were lower at baseline (*M* = −0.50, *SE* = 0.03) compared to post-stress (*M* = −0.42, *SE* = 0.02). However, this main effect was qualified by a significant Time × HC Use interaction predicting cortisol levels, *F*(1, 123) = 8.40, *p* =.004, $\eta_{\text{p}}^{2}=0.06$. Probing this interaction by examining the effect of HC Use on cortisol levels at each of the two time points (baseline and post-stress) revealed that, although cortisol levels did not differ between the two groups of women at baseline, *p* =.758, NC women had significantly lower levels of cortisol post-stress compared to women taking HCs, *p* =.016. Probing this interaction by examining the impact of Time on cortisol responses within each group of women (HC and NC) revealed that, although there were no differences between pre- and post-stress levels of cortisol for NC women, *p* =.705, women using HCs exhibited a significant increase in cortisol levels following stress, *p* ≤ 0.001. These results indicate that whereas NC women did not exhibit an increase in cortisol levels in response to stress, women taking HCs did.

#### 3.2.2. IL-1β Levels

A significant main effect of Time emerged, *F*(1, 122) = 19.78, *p* ≤ 0.001, $\eta_{\text{p}}^{2}=0.14$, with women having higher IL-1β levels at baseline (*M* = 1.69, *SE* = 0.06) compared to post-stress (*M* = 1.38, *SE* = 0.08). However, this main effect was qualified by a significant Time × HC Use interaction, *F*(1, 122) = 4.77, *p* =.031, $\eta_{\text{p}}^{2}=0.04$. Probing this interaction by examining the effect of HC Use on IL-1β levels at each of the two time points (baseline and post-stress) revealed that IL-1β levels did not differ between the two groups of women at either time point, *p*s ≥ 0.118. However, examining the effect of Time on IL-1β levels separately in each group of women revealed that women using HCs exhibited a significant decrease in IL-1β levels following stress, *p* ≤ 0.001, a pattern that was not observed among NC women (*p* =.103).

#### 3.2.3. IL-6 Levels

No significant effects of either HC Use or Time emerged, *p*s ≥ 0.507, indicating that neither HC nor NC women exhibited significant changes in IL-6 levels in response to the TSST.

#### 3.2.4. TNF-α Levels

A marginally significant main effect of Time on TNF-α levels emerged, *F*(1, 119) = 3.66, *p* =.058, $\eta_{\text{p}}^{2}=0.03$, with women having lower TNF-α levels at baseline (*M* = 0.07, *SE* = 0.04) compared to post-stress (*M* = 0.16, *SE* = 0.04). Additionally, a main effect of HC Use on TNF-α levels emerged, *F*(1, 119) = 7.55, *p* =.007, $\eta_{\text{p}}^{2}=0.06$, revealing that women using HCs exhibited higher levels of TNF-α across time points (*M* = 0.20, *SE* = 0.05) compared to NC women (*M* = 0.03, *SE* = 0.04). A significant Time × HC Use interaction did not emerge, *p* =.644.

### 3.3. Associations between Changes in Cortisol, Proinflammatory Cytokines, and Subjective Stress and Mood

After investigating differences in subjective and biological responses to stress between NC and HC-using women, change scores were computed to explore the associations between women’s biological and subjective stress responses, and whether the associations between these variables differed between these two groups of women. Change scores were computed by subtracting baseline values from post-stress values for each variable, per convention, with more positive scores indicating a greater increase in response to stress and more negative scores indicating a greater decrease in response to stress. See Table 4 for descriptive statistics of changes scores for each group of women. See Supplemental Materials (Tables S3–S5) for correlations between all biological change score variables and subjective change score variables, reported for the full sample and within each HC Use group.

Next, we examined whether (a) changes in cortisol predicted changes in proinflammatory cytokines and changes in women’s subjective responses to the stress manipulation and (b) these associations were moderated by women’s HC Use (dummy coded, NC = 0 vs HC = 1).

#### 3.3.1. Changes in IL-1β Levels

Results revealed a significant main effect of HC Use on changes in IL-1β, *b* = −0.44, *SE* = 0.15, *t* = 3.00, *p* =.003, indicating that women using HCs exhibited a larger decrease in IL-1β than did NC women in response to the TSST. A significant Changes in Cortisol × HC Use interaction predicting changes in IL-1β did not emerge, *b* = 0.82, *SE* = 0.56, *t* = 1.48, *p* =.143. See Fig. S1 for this non-significant interaction effect.

#### 3.3.2. Changes in IL-6

**Fig. 3.**
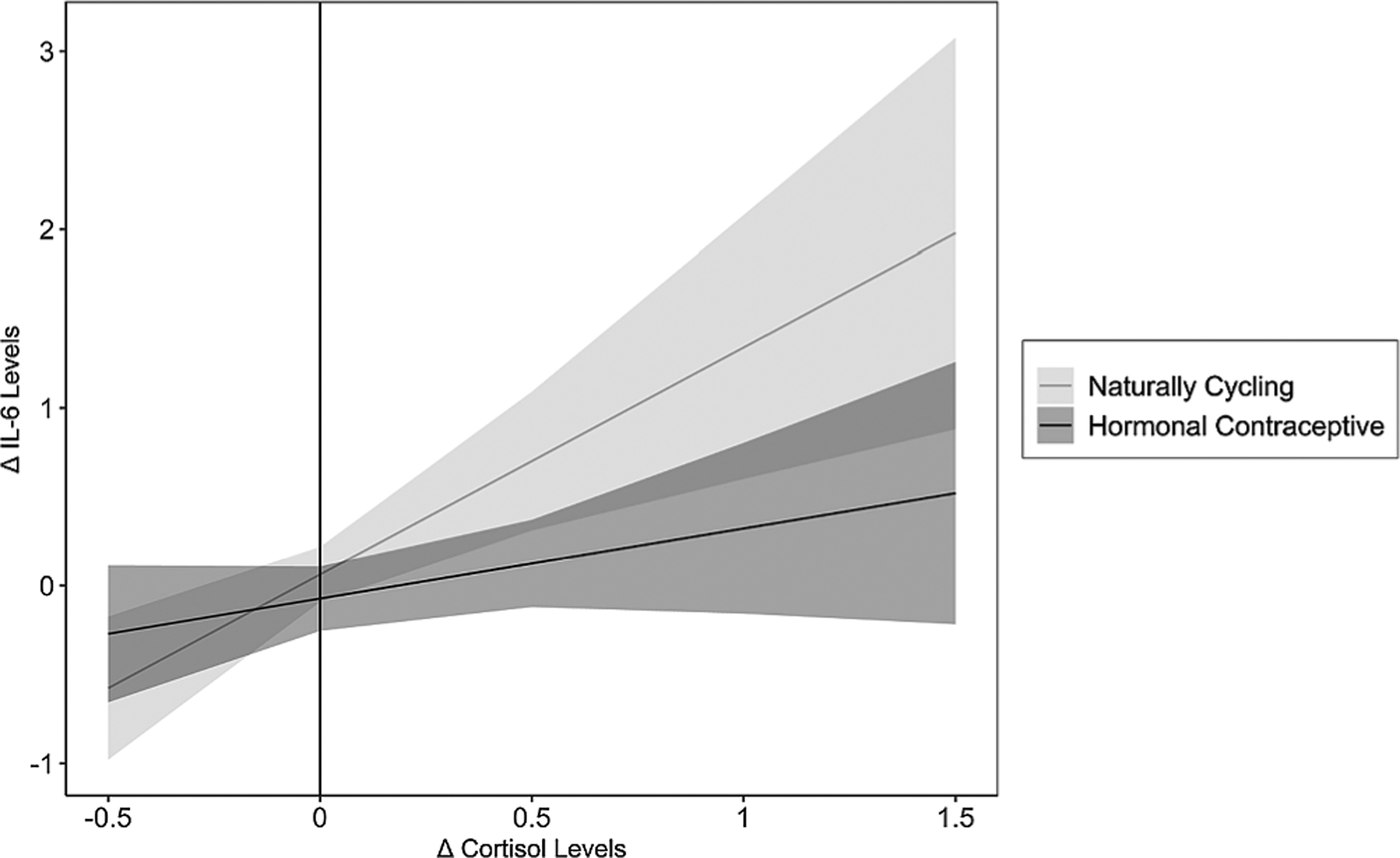
Association between Changes in Cortisol and changes in IL-6, moderated by Hormonal Contraceptive Use. In naturally cycling women, cortisol changes and changes in IL-6 were positively associated with each other, whereas this was not the case for hormonal contraceptives users. IL-6 = interleukin-6. Shadows indicate 95% confidence intervals.

Results revealed a significant main effect of changes in cortisol on changes in IL-6, *b* = 1.28, *SE* = 0.37, *t* = 3.50, *p* =.001, such that greater changes in cortisol predicted greater changes in IL-6 across both groups of women. However, this effect was qualified by a marginally significant Changes in Cortisol × HC Use interaction predicting changes in IL-6, *b* = −0.88, *SE* = 0.46, *t* = 1.93, *p* =.056. See Fig. 3 for interaction effect. Simple slopes tests revealed a significant positive relation between changes in cortisol and changes in IL-6 for NC women, *b* = 1.28, *SE* = 0.37, *t* = 3.47, *p* =.001, and no association between changes in cortisol and changes in IL-6 for women using HCs, *b* = 0.40, *SE* = 0.27, *t* = 1.46, *p* =.146.

Regions of significance testing revealed that there were no HC Use-based differences in the IL-6 response to the TSST when cortisol levels decreased in response to the TSST; however, when cortisol levels increased in response to the TSST (Changes in Cortisol ≥ 0.117, or ≥ 0.15 *SD*s above the mean of Changes in Cortisol), NC women exhibited a larger increase in IL-6 levels than did women using HCs, *p*s ≤ 0.050. Notably, only HC users with the largest increases in cortisol exhibited any increase in IL-6. Together, these results reveal that, for NC women, as cortisol levels increased, so too did their IL-6 levels, whereas this was not observed for women using HCs.

#### 3.3.3. Changes in TNF-α

**Fig. 4.**
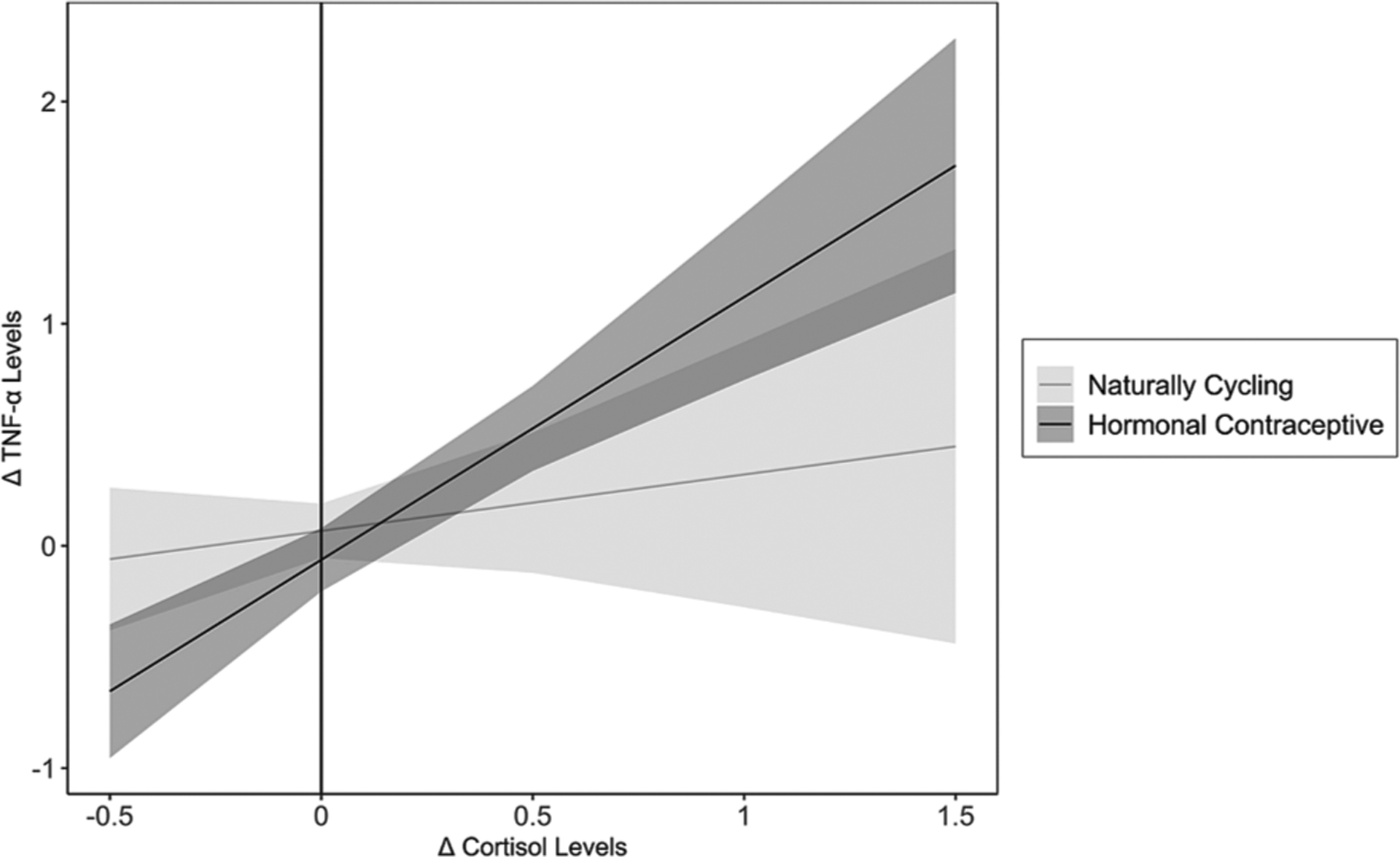
Association between Changes in Cortisol and changes in TNF-α, moderated by Hormonal Contraceptive Use. In women using hormonal contraceptives, cortisol changes and changes in TNF-α levels were positively associated with each other, whereas this was not the case for naturally cycling women. TNF-α = tumor necrosis factor-α. Shadows indicate 95% confidence intervals.

Results revealed a significant Changes in Cortisol × HC Use interaction predicting changes in TNF-α, *b* = 0.93, *SE* = 0.36, *t* = 2.56, *p* =.012. See Fig. 4.

Simple slopes analyses revealed a significant positive relation between changes in cortisol and changes in TNF-α for women using HCs, *b* = 1.18, *SE* = 0.21, *t* = 5.63, *p* ≤ 0.001, and no association between changes in cortisol and changes in TNF-α for NC women, *b* = 0.25, *SE* = 0.30, *t* = 0.85, *p* =.395. Regions of significance testing revealed HC use-based group differences when cortisol levels increased substantially in response to the TSST (Changes in Cortisol ≥ 0.62, or ≥ 2.01 *SD*s above the mean of Changes in Cortisol) on TNF-α change, with HC users having higher levels of TNF-α change compared to NC women, *p*s ≤ 0.050. Additionally, differences emerged between NC women and HC users when cortisol levels decreased in response to the TSST (Changes in Cortisol ≤ −0.09, or ≥ 0.60 *SD*s below the mean of Changes in Cortisol), *p*s ≤ 0.050. Here, women using HCs exhibited a decrease in TNF-α levels whereas NC women did not. These results reveal that for women using HCs, as cortisol levels increased, so too did their TNF-α levels, whereas this was not the case for NC women.

#### 3.3.4. Changes in Subjective Stress

Results revealed neither significant main effects of either HC Use or Changes in Cortisol, nor a significant Changes in Cortisol × HC Use interaction predicting changes in subjective stress, *p*s ≥ 0.335. These results reveal that changes in cortisol were not associated with changes in subjective stress for either group of women, nor did the association between these variables differ between NC women and women using HCs.

#### 3.3.5. Changes in Subjective Positivity of Mood

**Fig. 5.**
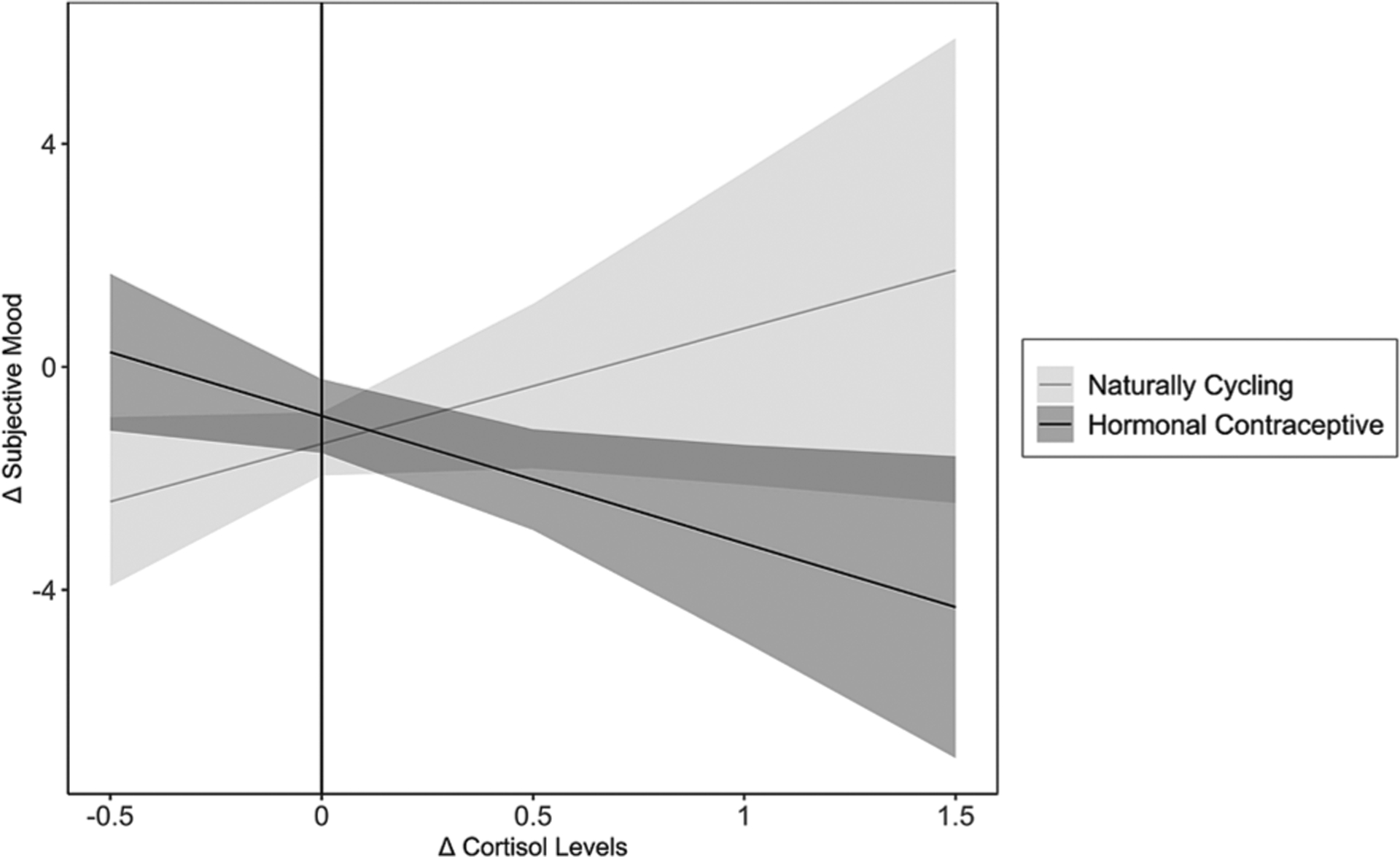
Association between Changes in Cortisol and changes in subjective positivity of mood, moderated by Hormonal Contraceptive Use. In women using hormonal contraceptives, changes in cortisol and changes in subjective positivity of mood were negatively associated with each other, whereas the opposite pattern emerged for naturally cycling women. Shadows indicate 95% confidence intervals.

Results revealed a significant Changes in Cortisol × HC Use interaction predicting changes in mood, *b* = −4.36, *SE* = 1.71, *t* = 2.55, *p* =.012. See Fig. 5. Simple slopes tests revealed a significant negative association between changes in cortisol and changes in mood for women using HCs, *b* = −2.29, *SE* = 0.99, *t* = 2.31, *p* =.023, and no association between changes in cortisol and changes in mood for NC women, *b* = 2.07, *SE* = 1.40, *t* = 1.48, *p* =.141. Investigating HC use-based differences in mood change revealed group differences when cortisol levels increased substantially in response to the TSST (Changes in Cortisol ≥ 0.54, or ≥ 1.71 *SD*s above the mean of Changes in Cortisol), *p*s ≤ 0.050, with HC users experiencing a larger decrease in the positivity of their mood compared to NC women. Additionally, differences emerged between NC women and HC users when cortisol levels decreased in response to the TSST (Changes in Cortisol ≤ −0.13, or ≥ 0.75 *SD*s below the mean of Changes in Cortisol), *p*s ≤ 0.050. When levels of cortisol decreased in response to the TSST, NC women exhibited a larger decrease in their subjective mood ratings than did women using HCs. These results reveal that changes in cortisol were associated with changes in subjective mood for women using HCs but not for NC women. Here, as cortisol levels increased following stress, women using HCs reported a more negative mood. For NC women, however, this pattern was reversed, although changes in cortisol and changes in subjective mood were not significantly associated with each other in NC women.

## 4. Discussion

A growing body of research has found that HC users exhibit more chronic inflammation (Larsen et al., 2020; Masama et al., 2022), oxidative stress (Cauci et al., 2021), and inflammation-related health conditions such as cardiovascular disease (Okoth et al., 2020), autoimmune disorders (Costenbader et al., 2007; Khalili et al., 2013), and depression (Skovlund et al., 2016) compared to NC women, despite the fact that proinflammatory cytokine levels generally do not differ between these groups (Caputi et al., 2022; Larsen et al., 2020). To investigate a possible explanation for this apparent paradox, we examined whether HC use predicted exaggerated inflammatory *reactivity* to an acute psychosocial stressor. We also examined the extent to which differences in women’s GC and inflammatory responses to stress predicted participants’ subjective appraisals of stress and mood following the stressor.

Contrary to prior research (Kirschbaum et al., 1999; Lovallo et al., 2019; Nielsen et al., 2013; Roche et al., 2013), we found that women using HCs exhibited a more robust, rather than blunted, cortisol response to the laboratory-based stressor than NC women. However, consistent with hypotheses, we found that users and non-users of HCs differed in their levels of inflammatory activity both before and in response to the TSST. Women using HCs had higher levels of TNF-α both before and after the stressor compared to NC women. Moreover, women using HCs exhibited a decrease in IL-1β levels in response to the stressor, which was not observed in NC women.

HC users and NC women also differed, somewhat, in the association between changes in their stress hormones and changes in inflammatory activity in response to the stressor. Specifically, changes in cortisol were positively related to changes in IL-6 for NC women, whereas changes in cortisol were positively related to changes in TNF-α for HC-using women. Furthermore, these latter differences predicted differences in women’s subjective responses to the acute stressor insofar as increases in cortisol were associated with more negative mood following the stressor for HC users, but not NC women. Together, these findings suggest that HC users may differ from non-users in terms of both their inflammatory response to acute stress and in how their inflammatory activity, cortisol, and mood are interrelated in such conditions. These findings may thus help explain the differences observed between NC women and HC users in levels of CRP (Divani et al., 2015; Larsen et al., 2020; Masama et al., 2022; c.f. Caputi et al., 2022; Eagan et al., 2021; Larsen et al., 2020) and risk of developing inflammation-related conditions, such as depression and autoimmune disease.

We also found that HC-using (but not NC) women exhibited a decrease in levels of IL-1β following the acute social stressor. Although not hypothesized, these patterns may arise from HC-related changes in HPA-axis reactivity to acute psychosocial stress. Consistent with this possibility, research has found that HC use predicts changes in HPA-axis activity that mimic those found for women exposed to chronic stress in early life (Hertel et al., 2017), which is a developmental context found to predict decreased circulating IL-1β levels following acute stress in rats (Roque et al., 2016).

In addition, we found that whereas changes in IL-6 were positively associated with changes in both cortisol and TNF-α for NC women, for HC users, changes in cortisol were positively associated with changes in both TNF-α and IL-1β, although this latter relation did not significantly differ for HC users vs. non-users. These results suggest that changes in social stressor-induced cortisol may promote different inflammatory responses in HC users vs. NC women. Although additional research is needed to examine whether this pattern replicates, if so, it could shed light on one mechanism underlying HC women’s increased risk of autoimmunity. Research has found that higher TNF-α levels play an important role in the development of certain autoimmune disorders (Jang et al., 2021). Given that HC users are at elevated risk of developing some autoimmune disorders compared to NC women (Costenbader et al., 2007; Khalili et al., 2013), it is possible that this increased risk is mediated through biasing women’s cytokine response to stress toward one marked by elevated levels of TNF-α. Future research is needed to examine this possibility and to better understand the mechanisms by which HC use impacts women’s inflammatory reactivity to stress.

Typically, GC and proinflammatory cytokine levels rise in response to acute stress, with the magnitude of these responses being inversely related (Kunz-Ebrecht et al., 2003). In addition, stress-induced changes in IL-6, TNF-α, and IL-1β are often similar to one another (Slavish et al., 2015). These patterns were not consistently observed here. Although, to our knowledge, no studies have investigated differential IL-6, TNF-α, and IL-1β responses to stress for NC vs. HC-using women, HC users appear to have a different profile of stress-induced inflammatory reactivity compared to NC women. These differences could emerge from the androgenic effects of HCs on the inflammatory response, with HC users having an inflammatory response to stress that is biased toward a more male-typical response. Consistent with this interpretation, research finds that women generally exhibit a larger but more delayed IL-6 response, and a smaller TNF-α response to stress than men (Edwards et al., 2006; Steptoe et al., 2002). Accordingly, the strong association between stress-induced changes in cortisol and IL-6 observed in NC women may represent a more “female-typical” inflammatory response, whereas the strong association between changes in cortisol and TNF-α observed for HC users may represent a more “male-typical” inflammatory response to acute social stress. If replicated, this finding could indicate that the androgenicity of HCs might be mechanistically responsible for shifting women’s inflammatory responses to acute social stress toward a more male-typical, TNF-α dominated, inflammatory response to social stress.

Few studies have investigated differences in women’s inflammatory response to stress at different ovulatory cycle phases or in relation to sex steroid hormone fluctuations. NC women in the present study were in their luteal cycle phase and expected to have high levels of progesterone compared to women using HCs. Although progesterone has known anti-inflammatory effects (Trzonkowski et al., 2001), the associations between sex-steroid hormones and inflammatory processes are nuanced and pleiotropic (see Gilliver, 2010). Despite this fact, fluctuating levels of progesterone and estradiol throughout the cycle likely impact NC women’s inflammatory responses to stress, potentially in opposite directions. Especially considering work finding that sex-steroid hormone levels moderate GC reactivity to acute stress (Barel et al., 2018; Juster et al., 2016), future research should explore the possibility that sex-steroid hormones and HC use may interact to predict women’s inflammatory responses to acute stress as well.

### 4.1. Associations Between Biological and Subjective Responses to Acute Stress

In the present study, we found a positive, although not significant, association between cortisol reactivity and ratings of subjective stress in both HC users and NC women. No association between cortisol reactivity and subjective stress reactivity was found in either group. However, changes in cortisol were differently associated with changes in mood following the acute stressor between NC women and HC users. Specifically, women using HCs reported significantly more negative mood in response to increased levels of cortisol following stress, whereas the opposite was observed for NC women. A similar association between changes in IL-6 and mood was also observed for both groups of women, with NC women exhibiting more positive moods alongside increases in IL-6 and HC using women exhibiting the opposite pattern. These results, whereby in NC women, GCs and IL-6 rise together in response to stress and are associated with a more positive mood, could reflect an effective stress-buffering response in NC women. In HC users, similar patterns are associated with a more negative mood after stress, which could indicate that HC use is associated with a biological stress response that may hamper women’s ability to cope effectively with stressors. Future research should explore these possibilities.

### 4.2. Unanticipated Results

Prior research has found that men and NC women in the luteal phase of their ovulatory cycles have a more pronounced GC response to stress than women using HCs and NC women in the follicular phase (Kirschbaum et al., 1999; Lovallo et al., 2019; Roche et al., 2013; Sharma et al., 2020). Here, however, we found that HC users exhibited a more robust GC response to acute stress than NC women in the luteal phase. Though unexpected, others have also found disparate results when investigating women’s GC responses to stress (Maki et al., 2015; Sharma et al., 2020). Although it is possible that our results are due to having assessed women in the luteal vs. follicular phase, or are driven by key unanalyzed moderators (e.g., age of HC onset), it is difficult to come to strong conclusions, as the last 30 years of research into associations between HC use and GC dysregulation have not yielded a clear mechanistic explanation for *how* HC impacts GC responses to stress.

In early work, Kirschbaum and colleagues (1999) suggested that sex differences in cortisol reactivity to stressors, and differences between NC women in different cycle phases and women using HCs, were likely attributable to differences in estradiol levels, the presence of exogenous estradiol, and levels of corticosteroid binding globulins, which bind to free cortisol, thereby lowering circulating levels of free cortisol. Since this landmark study was published, however, researchers have found mixed results when investigating associations between cortisol reactivity and estradiol levels. For example, controlling for levels of estradiol, progesterone, and testosterone results in some researchers reporting no differences in GC reactivity between men and NC women (Barel et al., 2018; Juster et al., 2016, in cortisol responders only). These results highlight the need to better understand how sex steroid hormones, which fluctuate across women’s ovulatory cycles and differ between NC women and women using HCs, impact GC reactivity, or if there are other key mechanistic processes driving the blunting of HC users’ GC responses to stress in past work which have yet to be discovered (see Rrapaj et al., 2023 for further discussion).

Likewise, it is possible that these findings resulted from the study taking place during a global pandemic (August 2021 and April 2022), during which time participants may have experienced chronic stress (Ayers et al., 2022; Charles et al., 2021; Kowal et al., 2020). Chronic, pandemic-related stress exposure, in turn, could have been responsible for the blunted cortisol responses to the acute stress task observed in NC women. Chronic stressor exposure has been found to blunt cortisol reactivity to acute stressors (Lam et al., 2019), potentially through adaptation of stress response systems (Coffman, 2020). As some have suggested that the HPA axis dysregulation typically observed in women using HCs is akin to what is observed in those who have experienced chronic stress (Hertel et al., 2017), the present results may indicate that under conditions of chronic stress, women using HCs exhibit a more robust cortisol response to stress than NC women. Future work could explore this possibility by investigating the extent to which chronic stressor exposure predicts acute stressor reactivity in HC and NC women.

### 4.3. Strengths and Limitations

Several limitations of this study should be noted. First, we tested all women before and after an acute social stressor, without a no-stress control group. Therefore, diurnal changes in cortisol may have yielded the appearance of no cortisol response to stress in our NC women, which would have been detectable if a no-stress control group was included. Based on past work finding women using HCs have an extended and exaggerated morning cortisol peak (Roche et al., 2013), diurnal changes in cortisol may have also yielded the appearance of a cortisol response to stress in our HC users, which would have not been observed if a no-stress control group was included. Second, all participants arrived at laboratory sessions after overnight fasting to limit variability in blood glucose levels, which can impact inflammation levels and cortisol reactivity to stress (Kirschbaum et al., 1997). Although this study design prevents variability in blood glucose levels from impacting inflammation levels, low blood glucose levels may have contributed to the relatively blunted cortisol reactivity to stress observed here (Kirschbaum et al., 1997). Third, we collected saliva samples for cortisol and inflammatory biomarkers at two time points: baseline and 30 min after onset of the stressor task. Although unlikely, we cannot rule out the possibility that stress hormones could have risen to detectable levels immediately and subsequently decreased by the time the post-stressor saliva sample was collected. Future research would benefit from collecting three or more salvia samples for up to two hours to capture the full rise and fall of social stressor-induced cortisol and proinflammatory cytokine levels (Slavish et al., 2015). Finally, as noted above, this study was conducted during a global pandemic, the chronic stress from which could have impacted these results in unmeasured ways.

Several strengths should also be noted. First, the study investigated both psychological and *in vivo* biological responses to an acute social stressor in both HC and NC women which, to our knowledge, has not previously been done. Second, we used a well-validated, laboratory-based social stressor, the TSST, to elicit a stress response from participants, which is critical for identifying individual differences in reactivity across individuals. Finally, the sample size was relatively large (*N* = 127) compared to prior studies on this topic, which should improve the reproducibility of these findings.

### 4.4. Future Directions

Looking forward, additional research is needed to investigate the mechanisms by which HC use affects sex steroid hormone levels and biological and psychological responses to stress. Indeed, if the androgenicity of progestins contained in HCs is responsible for women’s blunted GC responses to stress when using HCs, researchers should find large differences in the GC responses to stress between second and fourth generation HC users. If the synthetic estradiol component of combined HCs is driving this effect, researchers should find large differences in the GC response to stress between combined and progestin only oral HC users. Answering these mechanistic questions will require the collection of much larger samples and to overcome hurdles inherent in conducting research that considers women’s cycle phases. Likely, the use of well-controlled, prospective studies in which women’s stress responses are assessed before and after beginning HC treatment will be needed, along with complementary research using non-human animal models, to fully understand how HC use, and different types of HC use, might impact women’s GC, inflammatory, and psychological responses to acute stress.

Additional research is also needed to investigate the role of early-life and lifetime exposure to major stressors, especially interpersonal stressors, given that such stressors are associated with the age of menarche (Natsuaki et al., 2009), and as such, the age of HC use onset, as well as psychological and biological stress reactivity (Giletta et al., 2017; McLoughlin et al., 2022; Slavich et al., 2023). In addition, as the present study only included women using first-through-third generation oral HCs, and only investigated differences in effects based upon generation of HC used (see Supplemental Materials for generational analyses), future research would benefit from investigating how the androgenicity of the progestin used in different HCs impacts women’s stress responses, along with the dosage of synthetic hormones contained in the HC method, the method of HC administration, a women’s duration of HC use, how timing of administration of HCs pills in relation to stressor exposure impact stress responses, and the genomic mechanisms through which HC use impacts women’s stress responses. Beyond laying the groundwork for a better understanding of how different forms of HCs are associated with women’s health, overall well-being, and abilities to cope with stress, this research is vital for understanding the mechanisms by which HC use impacts women’s stress responses.

### 4.5. Conclusion

In conclusion, the present data are the first that we know of to characterize psychological and *in vivo* biological responses to an acute social stressor in both NC and HC-using women. For NC women, cortisol and IL-6 rose together in response to the stressor, and these biological responses were accompanied by more positive mood compared to women using HCs, suggesting a more adaptive response to acute stress. For women using HCs, in contrast, cortisol and TNF-α rose together in response to the stressor, and these biological responses were accompanied by more *negative* mood, indicating a less adaptive response. Future research is needed to investigate the mechanisms underlying these interactions and to translate these findings into improved mental and physical health for the millions of women who use HCs.

## Supplementary Material

Supplementary Material

## Figures and Tables

**Table 1:** Characteristics of the Data Analytic Sample (*N* = 127).

Variable	*M* (*SD*)
Age (Range: 18–37)	19.31 (1.95)
BMI (Range: 16.78–38.98)	21.94 (3.55)
Hormonal Contraceptive Use	
Naturally Cycling (*n* = 67)	
First Generation (*n* = 22)	
Second Generation (*n* = 11)	
Third Generation (*n* = 27)	
Race/Ethnicity	
White: 63.8% (*n* = 81)	
Black/African American: 3.1% (*n* = 4)	
Hispanic: 17.3% (n = 22)	
Asian/Pacific Islander: 6.3% (*n* = 8)	
Multiracial/Other: 9.5% (*n* = 12)	

*Note*. BMI = body mass index.

**Table 2:** Means and (Standard Deviations) of Subjective Responses to Stress across Time by Hormonal Contraceptive-Use Status.

	Baseline	Immediately Post-Stress	Post-Stress	End of Study
**Subjective Stress Levels**				
Naturally Cycling	4.29 (3.09)	4.98 (2.90)	4.13 (2.83)	3.55 (2.82)
Hormonal Contraceptive	5.04 (2.74)	6.09 (2.96)	5.48 (2.80)	4.65 (2.71)
**Positivity of Mood**				
Naturally Cycling	7.97 (2.14)	6.14 (2.62)	6.63 (2.36)	7.58 (2.26)
Hormonal Contraceptive	7.42 (1.89)	5.71 (2.11)	6.22 (2.00)	7.27 (1.71)

**Table 3:** Means and (Standard Deviations) for Cortisol and Inflammatory Biomarkers by Hormonal Contraceptive-Use Status.

	Transformed Values	
	Baseline	Post-Stress
**Naturally Cycling** (*n* = 63–65*)		
Cortisol	−0.49 (0.31)	−0.48 (0.30)
IL-1β	1.65 (0.76)	1.50 (0.76)
IL-6	0.36 (0.81)	0.42 (0.64)
TNF-α	−0.01 (0.39)	0.06 (0.47)
**Hormonal Contraceptive** (*n* = 58–60*)		
Cortisol	−0.50 (0.30)	−0.36 (0.21)
IL-1β	1.72 (0.66)	1.25 (0.95)
IL-6	0.43 (0.64)	0.41 (0.67)
TNF-α	0.14 (0.41)	0.26 (0.48)

Note. Values have been log transformed, a constant of 1 has been added to all values, and outliers trimmed to +/−3 standard deviations from the mean. Raw values with interpretable units can be found in Supplemental Materials Table S2. IL-1β = interleukin-1β; IL-6 = interleukin-6; TNF-α = tumor necrosis factor-α.

* Sample size (*n*) varies by analyte when assay values were out of range.

**Table 4:** Means and (Standard Deviations) for Change Scores by Hormonal Contraceptive Use Status.

	ΔCortisol	ΔIL-1β	ΔIL-6	ΔTNF-α	ΔStress	ΔMood
**Naturally Cycling**	0.01 (0.21)	−0.15 (0.65)	0.07 (0.70)	0.06 (0.43)	−0.08 (2.12)	−1.41 (2.38)
**Hormonal Contraceptive**	0.15 (0.31)	−0.46 (0.90)	−0.01 (0.61)	0.12 (0.65)	0.31 (1.98)	−1.23 (2.25)

Note. IL-1β = interleukin-1β; IL-6 = interleukin-6; TNF-α = tumor necrosis factor-α.

## Data Availability

Data will be made available on request.

## Footnotes

1 Five women with measured BMIs of greater than 30 were included in analyses. The pattern of results did not change when controlling for the effects of BMI on cortisol or inflammation levels.

2 Three participants were allowed to complete the in-person session with blood glucose levels higher than 105. The pattern of results did not change when controlling for the effects of blood glucose levels on cortisol or inflammation levels.

3 Although the manufacturer instructions recommend a 1:1 dilution factor, we used a 2:1 dilution factor to enable us to capture the low inflammation levels typical of healthy college students.
