# Supplementary material for: Hormonal contraceptive use is associated with differences in women’s inflammatory and psychological reactivity to an acute social stressor
Source: Brain Behav Immun. Author manuscript; Available in PMC 2024 Jul 1. (PMC11216059; doi:10.1016/j.bbi.2023.10.033)
Supplement: Supplementary Material [file NIHMS2005067-supplement-Supplementary_Material.docx]

Supplemental Materials for

**Hormonal Contraceptive Use is Associated with Differences in Women’s Inflammatory and Psychological Reactivity to an Acute Social Stressor**

Summer Mengelkoch, PhD^1,2^, Jeffrey Gassen, PhD^1^, George M. Slavich, PhD^1^, & Sarah E. Hill, PhD^2^

^1^ Department of Psychiatry and Biobehavioral Sciences, University of California, Los Angeles, 760 Westwood Plaza, Los Angeles, CA, 90095

^2^ Department of Psychology, Texas Christian University, 2955 South University Drive, Fort Worth TX, 76129

**Corresponding Author**: Summer Mengelkoch, Department of Psychiatry and Biobehavioral Sciences, University of California, Los Angeles, 760 Westwood Plaza, Los Angeles, CA, 90095. Email: [smengelkoch@mednet.ucla.edu](mailto:smengelkoch@mednet.ucla.edu)

**Table of Contents**

**Part 1: Supplemental Results** 3

Table S1: Skewness and Kurtosis Statistics for Cortisol and Inflammatory Biomarkers Before and After Transformations 3

Table S2: Means and (Standard Deviations) for Cortisol and Inflammatory Biomarkers Broken Down by HC-Use Status 3

Supplementary Associations Between Changes in Cortisol and Changes in IL-1β levels 4

Figure S1: Association between changes in cortisol and changes in IL-1β, moderated by hormonal contraceptive use4

Exploratory Correlations Between Changes in Responses to TSST5

Table S3: Correlations Between Change in Cortisol, Change in Inflammatory Biomarkers, and Change in Subjective Responses to the TSST 6

Table S4: Correlations Between Change in Cortisol, Change in Inflammatory Biomarkers, and Change in Subjective Responses to the TSST in Naturally Cycling Women 6

Table S5: Correlations Between Change in Cortisol, Change in Inflammatory Biomarkers, and Change in Subjective Responses to the TSST in Hormonal Contraceptive Users 7

Harmonic Mean *P*-Value Analysis8

Table S6a & S6b: Harmonic Mean *p*-value Analysis: All Test *p*-values & Results9

**Part 2: Results Reported by HC Generation** 11

Supplementary Data Analysis Plan 11

Supplementary Results: Differences Between Generations of HCs 13

Table S7: Means and (Standard Deviations) of Subjective Responses to Stress 13

Table S8: Means and (Standard Deviations) for Cortisol and Inflammatory Biomarkers, Reported by Generation of HC 16

Figure S2. Cortisol and inflammatory responses to the Trier Social Stress Test (TSST; reported with log-transformed values), Reported by Generation of Hormonal Contraceptive Use 17

Table S9: Means and (Standard Deviations) for Difference Scores, Reported by Generation of HC Use 20

Table S10: Means and (Standard Deviations) for Cortisol and Inflammatory Biomarkers, Reported by HC Generation: First Generation 20

Table S11: Means and (Standard Deviations) for Cortisol and Inflammatory Biomarkers, Reported by HC Generation: Second Generation 21

Table S12: Means and (Standard Deviations) for Cortisol and Inflammatory Biomarkers, Reported by HC Generation: Third Generation 22

Supplemental Discussion23

Table S13: Generations of HC Progestins 26

References 26

**Part 1: Supplemental Results**

| **Table S1**  *Skewness and Kurtosis Statistics for Cortisol and Inflammatory Biomarkers Before and After Transformations* | | | | | | |
| --- | --- | --- | --- | --- | --- | --- |
|  | **Raw Values** | | **Log Transformed Values** | | **Log Transformed and Windsorized Values** | |
|  | **Baseline** | **Post-Stress** | **Baseline** | **Post-Stress** | **Baseline** | **Post-Stress** |
| **Skewness** |  |  |  |  |  |  |
| Cortisol | 1.30 | 2.28 | -0.67 | -0.43 | -0.46 | -0.31 |
| IL-1β | 2.99 | 5.46 | -1.02 | -0.66 | -0.97 | -0.66 |
| IL-6 | 4.87 | 8.36 | 0.74 | 0.37 | 0.74 | 0.28 |
| TNF-α | 9.64 | 9.46 | 0.57 | 0.53 | 0.28 | 0.28 |
| **Kurtosis** |  |  |  |  |  |  |
| Cortisol | 2.70 | 8.05 | 0.92 | 1.26 | -0.01 | 0.84 |
| IL-1β | 11.34 | 40.60 | 1.09 | -0.36 | 0.88 | -0.36 |
| IL-6 | 26.06 | 78.92 | 0.94 | 1.46 | 0.94 | 1.16 |
| TNF-α | 101.40 | 97.56 | 1.23 | 1.90 | -0.10 | 0.93 |
| Note. Windsorized values have had outliers trimmed to +/-3 standard deviations from the mean; IL-1β = interleukin one beta; IL-6 = interleukin six; TNF-α = tumor necrosis factor alpha. | | | | | | |

| **Table S2**  *Means and (Standard Deviations) for Cortisol and Inflammatory Biomarkers Broken Down by HC-Use Status* | | |
| --- | --- | --- |
|  | **Raw Values** | |
|  | **Baseline** | **Post-Stress** |
| **Naturally Cycling** (*n* = 63-65*) | |  |
| Cortisol (μg/dL) | 0.41 (0.28) | 0.42 (0.29) |
| IL-1β (pg/mL) | 117.54 (171.44) | 81.79 (107.45) |
| IL-6 (pg/mL) | 19.75 (59.00) | 16.07 (63.94) |
| TNF-α (pg/mL) | 2.16 (7.00) | 2.28 (4.40) |
| **Hormonal Contraceptive** (*n* = 58-60*) | |  |
| Cortisol (μg/dL) | 0.38 (0.23) | 0.49 (0.29) |
| IL-1β (pg/mL) | 115.39 (139.00) | 88.98 (196.31) |
| IL-6 (pg/mL) | 9.67 (20.65) | 7.52 (14.75) |
| TNF-α (pg/mL) | 2.08 (1.88) | 4.93 (16.62) |
| Note. IL-1β = interleukin one beta; IL-6 = interleukin six; TNF-α = tumor necrosis factor alpha. *Sample size (*n*) varies by analyte when assay values were out of range. | | |

**Supplementary Associations Between Changes in Cortisol and Changes in IL-1β levels**

Results revealed a significant main effect of HC use on changes in IL-1β levels, *b* = -0.44, *SE* = .15, *t* = 3.00, *p* = .003, indicating that women using HCs exhibited a larger decrease in IL-1β levels vs. NC women in response to the Trier Social Stress Task (TSST). A significant two-way interaction between changes in cortisol levels and HC use on changes in IL-1β levels did not emerge, *b* = 0.82, *SE* = .56, *t* = 1.48, *p* = .143. See Figure S1 for non-significant interaction effect.

***

***

**Figure S1**. **Association between changes in cortisol and changes in IL-1β, moderated by hormonal contraceptive use.** In women using hormonal contraceptives, cortisol changes and changes in IL-1β were positively associated with each other, while this was not the case for naturally cycling women. However, a significant two-way interaction did not emerge.

IL-1β = interleukin one beta.

Although the interaction between changes in cortisol levels and HC use was not statistically significant, simple slope and regions of significance analyses are reported to better understand patterns of inflammatory responses to stress between naturally cycling (NC) women and women using HCs. Simple slopes analyses revealed a significant positive relationship between changes in cortisol levels and changes in IL-1β levels for women using HCs, *b* = 0.74, *SE* = .33, *t* = 2.25, *p* = .026, and no relationship between changes in cortisol levels and changes in IL-1β levels for NC women, *b* = -0.08, *SE* = .45, *t* = 0.18, *p* = .858. Investigating regions of significance testing revealed HC use-based differences in the IL-1β response to stress when cortisol levels either increased slightly, or when cortisol levels decreased in response to the TSST, (change in cortisol levels ≤ 0.165, or ≤ 0.33 *SD*s above the mean of cortisol change), with HC users having a larger decrease in IL-1β levels vs. NC women, *p*s ≤ .050. These results reveal that decreases in cortisol, were associated with a decrease in IL-1β levels for women using HCs, but not for NC women, although associations between changes in cortisol and changes in IL-1β levels were not significantly different between the groups, and should be interpreted with extreme caution.

**Exploratory Correlations Between Changes in Responses** **to TSST**

After conducting the target analyses, we explored the correlations between each of the measured physiological and subjective responses to the TSST separately for HC users and NC women. These post-facto, exploratory analyses were conducted to getter a better understanding of the associations between each of the measured changes in response to the TSST, and whether the patterns differ between users and HC users and NC women. See Table S3 for correlations between changes in cortisol and changes in inflammatory biomarkers and changes in subjective responses to the TSST, and see Table S4 and Table S5 for these correlations broken down by HC use status.

| **Table S3**  *Correlations Between Change in Cortisol, Change in Inflammatory Biomarkers, and Change in Subjective Responses to the TSST* | | | | | |
| --- | --- | --- | --- | --- | --- |
|  | Δ**IL-1β** | Δ**IL-6** | Δ**TNF-α** | **ΔStress** | **ΔMood** |
| **ΔCortisol** | *r* = .089  *n* = 123 | *r* = .222*  *n* = 123 | *r* = .414**  *n* = 120 | *r* = .032  *n* = 118 | *r* = -.081  *n* = 119 |
| **ΔIL-1β** |  | *r* = -.071  *n* = 124 | *r* = -.013  *n* = 121 | *r* = -.018  *n* = 119 | *r* = -.017  *n* = 120 |
| **ΔIL-6** |  |  | *r* = .227*  *n* = 121 | *r* = -.081  *n* = 119 | *r* = -.033  *n* = 120 |
| Δ**TNF-α** |  |  |  | *r* = .029  *n* = 117 | *r* = -.031  *n* = 118 |
| **ΔStress** |  |  |  |  | *r* = -.227*  *n* = 119 |
| Note. Δ = change; IL-1β = interleukin-1β; IL-6 = interleukin-6; TNF-α = tumor necrosis factor-α. ** *p* ≤ .001, * *p* ≤ .05 | | | | | |

| **Table S4**  *Correlations Between Change in Cortisol, Change in Inflammatory Biomarkers, and Change in Subjective Responses to the TSST in Naturally Cycling Women* | | | | | |
| --- | --- | --- | --- | --- | --- |
|  | **ΔIL-1β** | **ΔIL-6** | **ΔTNF-α** | **ΔStress** | **ΔMood** |
| **ΔCortisol** | *r* = -.027  *n* = 64 | *r* = .391**  *n* = 64 | *r* = .123  *n* = 62 | *r* = .011  *n* = 61 | *r* = .181  *n* = 62 |
| **ΔIL-1β** |  | *r* = .044  *n* = 65 | *r* = -.049  *n* = 63 | *r* = -.018  *n* = 62 | *r* = .193  *n* = 63 |
| **ΔIL-6** |  |  | *r* = .331*  *n* = 63 | *r* = -.154  *n* = 61 | *r* = .191  *n* = 63 |
| **ΔTNF-α** |  |  |  | *r* = -.153  *n* = 61 | *r* = .158  *n* = 62 |
| **ΔStress** |  |  |  |  | *r* = -.163  *n* = 62 |
| Note. Δ = change; IL-1β = interleukin-1β; IL-6 = interleukin-6; TNF-α = tumor necrosis factor-α; ** *p* ≤ .001, * *p* ≤ .05. | | | | | |

***Associations Between Changes in Physiological and Subjective Responses to the TSST: NC Women***

Results revealed that changes in cortisol were positively associated with changes in IL-6, *r*(64) = .391, *p* = .001. Additionally, changes in IL-6 were positively associated with changes in TNF-α, *r*(63) = .331, *p* = .008. No other associations between changes in stress responses reached significance in NC women, *p*s ≥ .129.

| **Table S5**  *Correlations Between Change in Cortisol, Change in Inflammatory Biomarkers, and Change in Subjective Responses to the TSST in Hormonal Contraceptive Users* | | | | | |
| --- | --- | --- | --- | --- | --- |
|  | **ΔIL-1β** | **ΔIL-6** | **ΔTNF-α** | **ΔStress** | **ΔMood** |
| **ΔCortisol** | *r* = .250  *n* = 59 | *r* = .198  *n* = 59 | *r* = .563**  *n* = 58 | *r* = .004  *n* = 57 | *r* = -.311*  *n* = 57 |
| **ΔIL-1β** |  | *r* = -.206  *n* = 59 | *r* = .060  *n* = 58 | *r* = -.013  *n* = 57 | *r* = -.112  *n* = 57 |
| **ΔIL-6** |  |  | *r* = .161  *n* = 58 | *r* = .025  *n* = 57 | *r* = -.174  *n* = 57 |
| **ΔTNF-α** |  |  |  | *r* = .164  *n* = 56 | *r* = -.184  *n* = 56 |
| **ΔStress** |  |  |  |  | *r* = -.318*  *n* = 57 |
| Note. Δ = change; IL-1β = interleukin-1β; IL-6 = interleukin-6; TNF-α = tumor necrosis factor-α; ** *p* ≤ .001, * *p* ≤ .05 | | | | | |

***Associations Between Changes in Physiological and Subjective Responses to the TSST: HC Users***

Results revealed that changes in cortisol were positively associated with changes in TNF-α, *r*(58) = .563, *p* ≤ .001, and negatively associated with changes in mood, *r*(57) = .311, *p* = .019. Additionally, changes in mood were negatively associated with changes in subjective stress levels, *r*(67) = .318, *p* = .016. No other associations between changes in stress responses reached significance in HC women.

**Harmonic Mean *P*-Value Analysis**

When multiple comparisons are made or when multiple hypotheses are tested, there is concern that the familywise error rate may be inflated to a degree that results in significant but spurious results. While this concern is valid under some circumstances (e.g., genome-wide association studies), the topic is more nuanced than is often discussed (e.g., Gelman et al., 2012; Lawrence, 2019; Perneger, 1998; Rothman, 1990; Rubin, 2017; Steegen et al., 2016). Inappropriately controlling for familywise error rate inflates Type II error rates and risks penalizing researchers for conducting comprehensive tests of a given hypothesis (in this case, examining multiple components of inflammation instead of a single component; Perneger, 1998; Rothman, 1990; Glickman et al., 2014). As most multiple comparison corrections assume independence of tests, they are poorly suited for addressing the concerns related to multiple comparisons in the current data, as many of the inflammation measures are indeed related (Gelman, 2012; Moran, 2003; Pena et al., 2011). Accordingly, if any of the null hypotheses are correctly rejected, the probabilities of false positives in the current work are relatively decreased compared to what the probabilities of false positives would be if the measures were independent (Frane, 2016). With these considerations in mind, we have utilized a recently developed, powerful tool to control for any increased familywise error rates given the dependent nature of our data.

In the current work, we conducted a harmonic mean *p*-value (HMP) analysis using R statistical software (R Core Team, 2021) in R Studio (R Studio Team, 2019) with the package *harmonicmeanp* (Wilson, 2019a; Wilson, 2019). HMP utilizes the harmonic mean of a set of *p*-values to produce an omnibus test of the null hypothesis (here, that there is no relationship between hormonal contraceptive use and stress responses) based on the number of tests conducted, and it is robust to dependency between *p*-values across tests.

The results of the analysis, reported in more detail in Table S6a-S6b, revealed that HMPs were significant when all original *p*-values were included (HMP: *p* = 0.001), when all tests of HC use were included (HMP: *p* = 0.041), when only those for effects on inflammation were included (HMP: *p* = 0.003), when only those for effects cortisol were included (HMP: *p* = 0.032), when only those for effects on subjective stress were included (HMP: *p* = 0.003), and when only those for effects positivity of mood were included (HMP: *p* = 0.003). Further, HMPs were significant when only tests for effects on change in inflammation were included (HMP: *p* = 0.003), but not when change only tests for effects on change in subjective stress (HMP: *p* = 1) or change in positivity of mood were included (HMP: *p* = 0.896). Together, these results support our overall interpretations, but suggest that the specific effects reported on change in positivity of mood should be interpreted with caution as these tests became non-significant after correcting for multiple hypothesis testing.

| **Table S6a**  *Harmonic Mean p-value Analysis: All Test p-values* | | |
| --- | --- | --- |
| **Predictor** | **Dependent Measure** | ***p*** |
| HC use | Cortisol Levels | 0.286 |
| Time | Cortisol Levels | 0.001 |
| HC use * Time | Cortisol Levels | 0.004 |
| HC use | TNF-α Levels | 0.007 |
| Time | TNF-α Levels | 0.058 |
| HC use * Time | TNF-α Levels | 0.644 |
| HC use | IL-6 Levels | 0.78 |
| Time | IL-6 Levels | 0.652 |
| HC use * Time | IL-6 Levels | 0.507 |
| HC use | IL-1β Levels | 0.47 |
| Time | IL-1β Levels | 0.0001 |
| HC use * Time | IL-1β Levels | 0.031 |
| HC use | Subjective Stress | 0.028 |
| Time | Subjective Stress | 0.0001 |
| HC use * Time | Subjective Stress | 0.474 |
| HC use | Positivity of Mood | 0.213 |
| Time | Positivity of Mood | 0.0001 |
| HC use * Time | Positivity of Mood | 0.922 |
| HC use | Δ TNF-α Levels | 0.173 |
| Δ Cortisol Levels | Δ TNF-α Levels | 0.394 |
| HC use * Δ Cortisol Levels | Δ TNF-α Levels | 0.012 |
| HC use | Δ IL-6 Levels | 0.264 |
| Δ Cortisol Levels | Δ IL-6 Levels | 0.001 |
| HC use * Δ Cortisol Levels | Δ IL-6 Levels | 0.056 |
| HC use | Δ IL-1β Levels | 0.003 |
| Δ Cortisol Levels | Δ IL-1β Levels | 0.858 |
| HC use* Δ Cortisol Levels | Δ IL-1β Levels | 0.143 |
| HC use | Δ Subjective Stress | 0.355 |
| Δ Cortisol Levels | Δ Subjective Stress | 0.982 |
| HC use* Δ Cortisol Levels | Δ Subjective Stress | 0.953 |
| HC use | Δ Positivity of Mood | 0.258 |
| Δ Cortisol Levels | Δ Positivity of Mood | 0.141 |
| HC use * Δ Cortisol Levels | Δ Positivity of Mood | 0.012 |
| Note. HC = hormonal contraceptive; Δ = change; IL-1β = interleukin-1β; IL-6 = interleukin-6; TNF-α = tumor necrosis factor-α; | | |

| **Table S6b**  *Harmonic Mean P-Value Analysis Results* | |
| --- | --- |
| Weights (w): equal; Total tests (L): 33 | |
|  |  |
| ***p*-values Tested** | **Harmonic mean *p*-values *(HMPs)*** |
| All tests (total) | *p* = 0.001009 |
| HC use | *p* = 0.04104 |
| Inflammation | *p* = 0.003389 |
| Cortisol | *p* = 0.03210 |
| Subjective Stress | *p* = 0.003389 |
| Positivity of Mood | *p* = 0.00334 |
| Δ Inflammation | *p* = 0.029114 |
| Δ Subjective Stress | *p* = 1 |
| Δ Positivity of Mood | *p* = 0.8956 |
| Note. Weights (w): equal; Total tests (L): 33. HC = hormonal contraceptive; Δ = change. | |

**Part 2: Results Reported by HC Generation**

Historically, stress researchers have treated all women taking HCs as one homogenous group, with little attention paid to the type of hormonal contraceptive (HC) that women are using. However, a recent study by Herrera and colleagues (2019) is an exception. This team conducted the first study to better understand the differences in the cortisol responses to acute social stress in women depending upon the type of oral HC participants were taking. The researchers found that women taking HCs containing second generation progestins exhibited a larger cortisol response to stress compared to women taking HCs containing first or third generation progestins, who displayed more blunted cortisol responses to acute stress. These results highlight the possibility that some types of HCs could have a stronger influence on women’s cortisol response to stress than do others.

Building upon this work, an exploratory aim of the current project was to examine whether biological and subjective responses to stress in women using HCs vary as a function of the generation of progestin contained in the HC product they are using. Here, we began to explore whether there are differences in women’s biological and subjective responses to an acute psychosocial stressor depending on whether they were taking HCs containing first, second, or third generation progestins. These specific generations of oral HC were chosen for inclusion in the current study based upon previous work, which reports the largest differences in cortisol responses to stress between these generations of HCs (Herrera et al., 2019^^[[1]](#footnote-1)^^). Additionally, these first three generations of oral HCs were among the most commonly prescribed HCs at the time of data collection for the current study (Brynhildsen, 2014; Hall & Trussell, 2012). As such, investigating differences in women’s biological and subjective responses to stress between users of first, second, and third generation oral HCs will provide information that pertains to a large number of women and is an important first step towards beginning to understand the how different types of HC use are related to women’s inflammatory stress responses.

**Supplementary Data Analysis Plan**

We performed exploratory analyses specifically conducted within HC users, to determine if women using different generations of oral HCs display different subjective responses to stress, using a series of 4 (within-subjects Time: baseline vs. immediately post-stress vs. post-stress vs. end of study) X 3 (between-subjects HC generation: first vs. second vs. third generation HC users) mixed model Analysis of Variance (ANOVA) tests on subjective appraisals of stress and subjective positivity of mood. We then performed additional exploratory analyses to determine if women taking different generations of oral HCs display different biological responses to stress, using a series of mixed model 2 (within-subjects Time: baseline vs. post-stress) X 3 (between-subjects HC generation: first vs. second vs. third generation HC users) mixed model ANOVAs on cortisol and inflammatory biomarkers. While results of these analyses are largely nonsignificant, and analyses underpowered, trending simple effects (*p*s ≤ .150) were explored to begin to examine how different generations of HCs impact women’s cortisol and inflammatory responses to stress. Finally, we report correlations between changes in cortisol levels, changes in inflammatory biomarkers, changes in subjective appraisals of stress levels, and changes in subjective positivity of mood for each generation of HC users to begin to explore how these variables relate to each other for each generation of HC users. However, due to power constraints, we did not conduct additional moderated regression analyses between HC generation groups.

**Supplementary Results**

**Differences Between Generations of HCs**

***Subjective Responses to Stress***

See Table S7 for descriptive statistics for subjective responses to stress, reported by HC generation.

| **Table S7**  *Means and (Standard Deviations) of Subjective Responses to Stress* | | | | |
| --- | --- | --- | --- | --- |
|  | **Baseline** | **Immediately Post-Stress** | **Post-Stress** | **End of Study** |
| **Stress Levels** | | | | |
| Naturally Cycling | 4.29 (3.09) | 4.98 (2.90) | 4.13 (2.83) | 3.55 (2.82) |
| First Generation | 5.39 (2.29) | 6.21 (2.73) | 5.70 (2.74) | 4.71 (2.74) |
| Second Generation | 3.40 (2.88) | 4.49 (3.04) | 3.74 (3.23) | 3.51 (3.02) |
| Third Generation | 5.33 (2.95) | 6.59 (3.05) | 5.95 (2.53) | 5.05 (2.55) |
| **Positivity of Mood** | | | | |
| Naturally Cycling | 7.97 (2.14) | 6.14 (2.62) | 6.63 (2.36) | 7.58 (2.26) |
| First Generation | 7.12 (1.49) | 5.39 (2.12) | 5.75 (2.12) | 6.87 (1.69) |
| Second Generation | 7.76 (1.80) | 6.86 (2.18) | 7.07 (2.39) | 7.59 (2.50) |
| Third Generation | 7.57 (2.24) | 5.58 (2.00) | 6.34 (1.65) | 7.53 (1.32) |

**Subjective Stress Levels.** A mixed-model 4 (within-subjects Time: baseline vs. immediately post-stress vs. post-stress, vs. end of study) X 3 (between-subjects HC generation: first generation vs. second generation vs. third generation) ANOVA was performed on subjective, self-reported stress levels. A significant main effect of Time on subjective stress levels emerged, *F*(3, 153) = 7.75, *p* ≤ .001, η_p_^2^ = .13. Simple effect analyses revealed that participants reported the highest levels of subjective stress immediately following the stress task (*M* = 5.76, *SE* = 0.44) compared to what they reported at all other time points, *p*s ≤ .005. Additionally, participants reported higher levels of subjective stress post-stress (*M* = 5.13, *SE* = 0.41) compared to what they reported at the end of the study (*M* = 4.42, *SE* = 0.40), *p* = .015. Participants reported the same amount of stress at baseline (*M* = 4.84, *SE* = 0.40) compared to what they reported at all other time points, *p*s ≥ .161. Additionally, a trending main effect of HC generation on subjective stress levels emerged, *F*(2, 51) = 2.10, *p* = .113, η_p_^2^ = .08. Simple effect analyses revealed that second generation HC users (*M* = 3.79, *SE* = 0.83) reported lower levels of subjective stress throughout the study compared to first (*M* = 5.50, *SE* = 0.53) and third generation (*M* = 5.73, *SE* = 0.52) HC users, *p*s ≤ .087, who did not differ from each other, *p* = .756. A significant two-way interaction between Time and HC use did not emerge, *p* = .937. However, follow-up simple effect analyses investigating the impact of HC generation at each time point revealed that second generation HC users reported somewhat less subjective stress levels than both first and third generation users at baseline and post-stress, *p*s ≤ .078, and somewhat less stress than third generation HC users immediately post-stress, *p* = .074, but did not differ from first generation HC users at this time point, *p* = .142. First and third generation HC users did not differ from each other at any time point, *p*s ≥ .668, and there were no differences between users of different generations of HCs at the end of the study, *p*s ≥ .153. Additional follow-up simple effect analyses investigated the impact of Time within each HC generation, and revealed that women using first generation HCs reported lower stress levels at the end of the study compared to immediately post-stress or post-stress, *p*s ≤ .017. Baseline stress levels for women using first generation HCs did not differ from stress levels reported at any other time point, *p*s ≥ .100, and immediately post-stress subjective stress levels were marginally higher than post-stress stress levels, *p* = .084. Second generation HC users reported marginally no differences in their stress levels throughout the study, *p*s ≥ .112. Finally, women using third generation HCs reported more subjective stress immediately post-stress compared to all other time points, *p*s ≤ .032, more stress post-stress than at the end of the study, *p* = .027, and reported no other differences in subjective stress levels, *p*s ≥ .144. This pattern of results indicates that women using second generation HCs experienced less subjective stress throughout the course of the study than did women using first or third generation HCs.

**Subjective Positivity of Mood.** A mixed-model 4 (within-subjects Time: baseline vs. immediately post-stress vs. post-stress, vs. end of study) X 3 (between-subjects HC generation: first generation vs. second generation vs. third generation) ANOVA was performed on subjective, self-reported positivity of mood. A significant main effect of Time on subjective mood emerged, *F*(3, 153) = 15.22, *p* ≤ .001, η_p_^2^ = .23. Simple effect analyses revealed that participants reported a more positive mood at baseline (*M* = 7.48, *SE* = 0.28) compared to what they reported immediately post-stress (*M* = 5.94, *SE* = 0.31) and post-stress (*M* = 6.38, *SE* = 0.30), *p*s ≤ .002, which did not differ from their reported mood at the end of the study (*M* = 7.33, *SE* = 0.25), *p* = .566. Additionally, participants reported a more negative mood immediately post-stress compared to what they reported post-stress and what they reported at the end of the study, *p*s ≤ .043. Neither a significant main effect of HC generation nor a significant two-way interaction between Time and HC use on subjective mood emerged, *p*s ≥ .240.

Follow-up simple effect analyses investigating the impact of HC generation at each time point revealed that women using second generation HCs reported a somewhat more positive mood than did women using first generation HCs both immediately post-stress and post-stress, *p*s ≤ .099. No differences in mood between different generations of HC users at any time point emerged, *p*s ≥ .124. Additional follow-up simple effect analyses investigated the impact of Time within each HC generation on subjective mood, and revealed that women using first and third generation HCs reported a more positive mood at baseline and at the end of the study compared to both immediately post-stress and post-stress, *p*s ≤ .012, while the positivity of their mood did not differ between baseline and at the end of the study, *p*s ≥ .506, nor did it differ between immediately post-stress and post stress, *p* = .241, for first generation HC users. Third generation HC users, on the other hand, reported a more positive mood post-stress compared to what they reported immediately post-stress, *p* = .015. Finally, women using second generation HCs did not report differences in the positivity of their moods between any time points of the study, *p*s ≥ .197. This pattern of results indicates that while women using first and third generation HCs experienced a more negative mood following the stress task, that women using second generation HCs retained a more positive mood throughout the course of the study than did the other generations of HC users.

***Biological Responses to Stress***

See Table S8 and Figure S2 for descriptive statistics for biological responses to stress, broken down by HC generation.

| **Table S8**  *Means and (Standard Deviations) for Cortisol and Inflammatory Biomarkers, Reported by Generation of HC Use* | | | | | | |
| --- | --- | --- | --- | --- | --- | --- |
|  | **First Generation HC** | | **Second Generation HC** | | **Third Generation HC** | |
|  | **Baseline** | **Post-Stress** | **Baseline** | **Post-Stress** | **Baseline** | **Post-Stress** |
| **Cortisol** | -0.53 (0.34) | -0.40 (0.20) | -0.43 (0.28) | -0.33 (0.20) | -0.51 (0.28) | -0.34 (0.22) |
| **IL-1β** | 1.75 (0.74) | 1.03 (1.02) | 1.24 (0.64) | 0.75 (0.80) | 1.90 (0.50) | 1.66 (0.80) |
| **IL-6** | 0.50 (0.65) | 0.55 (0.58) | 0.62 (1.02) | 0.58 (1.07) | 0.28 (0.38) | 0.22 (0.49) |
| **TNF-α** | 0.22 (0.41) | 0.33 (0.36) | 0.27 (0.44) | 0.29 (0.72) | 0.03 (0.39) | 0.18 (0.47) |
| ***n*** | 22 | | 10-11 | | 26-27 | |
| Note. Values have been log transformed, and outliers trimmed to +/-3 standard deviations from the mean; HC = hormonal contraceptive; IL-1β = interleukin one beta; IL-6 = interleukin six; TNF-α = tumor necrosis factor alpha. | | | | | | |

**Figure S2**. **Cortisol and inflammatory responses to the Trier Social Stress Test (TSST; reported with log-transformed values), Reported by Generation of Hormonal Contraceptive Use.** Compared to users of other HC generations, 3^rd^ generation HC users had a larger cortisol response to the TSST, heighted IL-1β levels that did not decrease in response to the TSST, and low baseline levels of TNF-α. Note. 1^st^ = first generation HC users; 2^nd^ = second generation HC users; 3^rd^ = third generation HC users; IL-1β = interleukin one beta; IL-6 = interleukin six; TNF-α = tumor necrosis factor alpha.

**Cortisol Levels.** Results revealed a significant main effect of Time on cortisol levels, *F*(1, 57) = 10.68, *p* = .002, η_p_^2^ = .16. Pairwise comparisons revealed that women on all generations of HCs experienced a rise in cortisol levels post-stress (*M* = -0.36, *SE* = 0.03) compared to at baseline (*M* = -0.49, *SE* = 0.04). HC generation did not significantly predict cortisol levels, nor was there a significant two-way interaction between Time and HC generation on cortisol levels, *p*s ≥ .512. Simple effect analyses revealed, however, that first and third generation HC users experienced an increase in cortisol levels following stress, *p*s ≤ .039, while second generation users exhibited no significant difference between their cortisol levels before and after stress, *p* = .283. No other simple effects comparisons approached significance, *p*s ≥ .318. These results are suggestive of a cortisol response to stress being more pronounced in first and third generation HC users than second generation HC users, in contrast to what was found by Herrera and colleagues (2019).

**IL-1β Levels.** Results revealed a significant main effect of Time on IL-1β levels, *F*(1, 56) = 14.95, *p* ≤ .001, η_p_^2^ = .21. Pairwise comparisons revealed that levels of IL-1β decreased from baseline levels (*M* = 1.63, *SE* = 0.09) following stress (*M* = 1.15, *SE* = 0.12). Additionally, a main effect of HC generation emerged, *F*(2, 56) = 6.50, *p* = .003, η_p_^2^ = .19. Pairwise comparisons revealed that women using third generation HCs (*M* = 1.78, *SE* = 0.12) had significantly higher levels of IL-1β than did users of both first (*M* = 1.39, *SE* = 0.13) and second generation HCs (*M* = 1.00, *SE* = 0.19), *p*s ≤ .035, while first generation HC users had somewhat higher levels of IL-1β than did second generation HC users, *p* = .096. A significant two-way interaction did not emerge between Time and HC generation on IL-1β levels, *p* = .186. Simple effect analyses investigating differences between HC generations at each time point revealed that, at baseline, second generation HC users had significantly lower levels of IL-1β compared to both first and third generation HC users, *p*s ≤ .032, while users of first and third generation HCs did not differ from each other in IL-1β levels, *p* = .408. Additionally, following stress, third generation HC users had significantly higher levels of IL-1β compared to both first and second generation HC users, *p*s ≤ .017, while first and second generation HC users did not differ from each other, *p* = .403. Simple effect analyses investigating the effect of Time within each HC generation revealed that first generation users experienced a significant decrease in IL-1β levels following stress, *p* ≤ .001, while second generation users experienced a marginal decrease in IL-1β levels following stress, *p* = .075. Third generation HC users, however, exhibited no differences between their baseline and post-stress levels of IL-1β, *p* = .181. These results suggest that users of third generation HCs experience higher levels of IL-1β that do not decrease in response to stress, in comparison with first and second generation HC users.

**IL-6 Levels.** A marginally significant effect of HC generation on IL-6 levels emerged, *F*(2, 56) = 2.08, *p* = .134, η_p_^2^ = .07. Pairwise comparisons revealed that women using third generation HCs (*M* = 0.25, *SE* = 0.11) had somewhat lower levels of IL6 compared to women using first (*M* = 0.53, *SE* = 0.12) or second generation HCs (*M* = 0.60, *SE* = 0.17), *p*s ≤ .101, who did not differ from each other in IL6 levels, *p* = .724. Neither a main effect of Time, nor a two-way interaction between HC generation and Time on IL-6 levels approached significance, *p*s ≥ .826. Simple effect analyses revealed that women’s IL-6 levels did not change in response to stress in any generation of HC user, *p*s ≥ .639. Additional follow-up simple effect analyses revealed that third generation HC users had somewhat lower levels of IL-6 than did first or second generation HC users following stress, and somewhat lower levels of IL-6 than second generation HC users at baseline, *p*s ≤ .144. No other differences in IL-6 levels emerged between generations of HC users at either time point, *p*s ≥ .234. These results suggest that third generation HC users have lower levels of IL-6 when compared to first and third generation HC users, although results are not significant and should be interpreted with extreme caution.

**TNF-α Levels.** A marginally significant effect of HC generation on TNF-α levels emerged, *F*(2, 55) = 2.02, *p* = .143, η_p_^2^ = .07. Pairwise comparisons revealed that women using third generation HCs (*M* = 0.11, *SE* = 0.06) had somewhat lower levels of TNF-α compared to women using first (*M* = 0.27, *SE* = 0.07) or second generation HCs (*M* = 0.28, *SE* = 0.10), *p*s ≤ .145, who did not differ from each other in TNF-α levels, *p* = .940. Neither a main effect of Time, nor a two-way interaction between Time and HC generation on TNF-α levels approached significance, *p*s ≥ .295. Follow-up simple effect analyses revealed that women’s TNF-α levels did not change in response to stress in any generation of HC user, *p*s ≥ .230. Additional follow-up simple effect analyses revealed that third generation HC users had somewhat lower levels of TNF-α at baseline, *p*s ≤ .127. No other differences in TNF-α levels emerged between generations of HC users at either time point, *p*s ≥ .283. These results suggest that third generation HC users have lower baseline levels of TNF-α when compared to first and third generation HC users, although results are not traditionally significant and should be interpreted with caution.

***Correlations Between Changes in Responses to Stress***

To better understand how changes in biological and subjective responses to stress differ between women using different generations of HCs, we explored correlational relationships between changes in these variables. See Table S9 for descriptive statistics of these change scores for each generation of HC users (descriptive statistics for NC women are included for reference). See Tables S10, S11, and S12 for correlational relationships between changes in biological and subjective responses to stress for first, second, and third generation HC users, respectively.

| **Table S9**  *Means and (Standard Deviations) for Difference Scores, Reported by Generation of HC Use* | | | | | | |
| --- | --- | --- | --- | --- | --- | --- |
|  | **ΔCortisol** | **ΔIL-1β** | **ΔIL-6** | **ΔTNF-α** | **ΔStress** | **ΔMood** |
| **NC** | 0.01 (0.21) | -0.15 (0.65) | 0.07 (0.70) | 0.06 (0.43) | -0.08 (2.12) | -1.41 (2.38) |
| **1^st^ Gen** | 0.13 (0.29) | -0.72 (1.03) | 0.05 (0.53) | 0.12 (0.40) | 0.30 (1.64) | -1.37 (1.87) |
| **2^nd^ Gen** | 0.10 (0.18) | -0.49 (0.67) | -0.04 (0.84) | 0.02 (1.09) | 0.11 (1.19) | -1.01 (1.95) |
| **3^rd^ Gen** | 0.18 (0.36) | -0.24 (0.84) | -0.06 (0.28) | 0.17 (0.62) | 0.40 (2.50) | -1.18 (2.70) |
| Note. NC = naturally cycling; gen = generation; IL-1β = interleukin one beta; IL-6 = interleukin six; TNF-α = tumor necrosis factor alpha. | | | | | | |

| **Table S10**  *Correlations Between Change in Cortisol and Change in Inflammatory Biomarkers, Reported by HC Generation: First Generation* | | | | | | |
| --- | --- | --- | --- | --- | --- | --- |
|  | **ΔIL-1β** | **ΔIL-6** | **ΔTNF-α** | **ΔStress** | **ΔMood** |  |
| **ΔCortisol** | *r* = .069  *n* = 22 | *r* = .586*  *n* = 22 | *r* = .474*  *n* = 22 | *r* = -.097  *n* = 22 | *r* = -.300  *n* = 22 |  |
| **ΔIL-1β** |  | *r* = -.180  *n* = 22 | *r* = -.410  *n* = 22 | *r* = -.140  *n* = 22 | *r* = .145  *n* = 22 |  |
| **ΔIL-6** |  |  | *r* = .454*  *n* = 22 | *r* = -.132  *n* = 22 | *r* = -.427*  *n* = 22 |  |
| **ΔTNF-α** |  |  |  | *r* = .282  *n* = 22 | *r* = .035  *n* = 22 |  |
| **ΔStress** |  |  |  |  | *r* = .037  *n* = 22 |  |
| Note. NC = naturally cycling; HC = hormonal contraceptive; IL-1β = interleukin one beta; IL-6 = interleukin six; TNF-α = tumor necrosis factor alpha; ** *p* ≤ .001, * *p* ≤ .05. | | | | | | |

In first generation HC users, changes in cortisol levels were significantly positively correlated with changes in IL-6 and changes in TNF-α levels, and changes in IL-6 and changes in TNF-α levels were significantly positively correlated with each other as well. Additionally, changes in IL-6 levels were significantly negatively correlated with changes in subjective positivity in mood. Overall, when first generation HC users experienced a rise in cortisol, this was accompanied by rises in IL-6 and TNF-α levels, and these rises were accompanied by a more negative mood.

| **Table S11**  *Correlations Between Change in Cortisol and Change in Inflammatory Biomarkers, Reported by HC Generation: Second Generation* | | | | | | |
| --- | --- | --- | --- | --- | --- | --- |
|  | **ΔIL-1β** | **ΔIL-6** | **ΔTNF-α** | **ΔStress** | **ΔMood** |  |
| **ΔCortisol** | *r* = -.442  *n* = 11 | *r* = -.063  *n* = 11 | *r* = .587†  *n* = 10 | *r* = .035  *n* = 10 | *r* = -.423  *n* = 10 |  |
| **ΔIL-1β** |  | *r* = -.725*  *n* = 11 | *r* = -.260  *n* = 10 | *r* = -.163  *n* = 10 | *r* = -.147  *n* = 10 |  |
| **ΔIL-6** |  |  | *r* = .181  *n* = 10 | *r* = .411  *n* = 10 | *r* = -.005  *n* = 10 |  |
| **ΔTNF-α** |  |  |  | *r* = .449  *n* = 9 | *r* = -.003  *n* = 9 |  |
| **ΔStress** |  |  |  |  | *r* = .223  *n* = 10 |  |
| Note. NC = naturally cycling; HC = hormonal contraceptive; IL-1β = interleukin one beta; IL-6 = interleukin six; TNF-α = tumor necrosis factor alpha; ** p ≤ .001, * p ≤ .05 † p ≤ .074. | | | | | | |

In second generation HC users, changes in cortisol levels following stress were marginally significantly positively correlated with changes in TNF-α levels. Additionally, changes in IL-1β levels were significantly negatively correlated with changes in IL-6 levels. These results highlight that when second generation HC users experienced a rise in cortisol in response to stress, this rise was accompanied by a rise in TNF-α levels. Additionally, when they experienced a decrease in IL-1β levels, this decrease was accompanied by a rise in IL-6 levels.

| **Table S12**  *Correlations Between Change in Cortisol and Change in Inflammatory Biomarkers, Reported by HC Generation: Third Generation* | | | | | | |
| --- | --- | --- | --- | --- | --- | --- |
|  | **ΔIL-1β** | **ΔIL-6** | **ΔTNF-α** | **ΔStress** | **ΔMood** |  |
| **ΔCortisol** | *r* = .491*  *n* = 26 | *r* = .075  *n* = 26 | *r* = .727**  *n* = 26 | *r* = .042  *n* = 25 | *r* = -.310  *n* = 25 |  |
| **ΔIL-1β** |  | *r* = .019  *n* = 26 | *r* = .536*  *n* = 26 | *r* = .084  *n* = 25 | *r* = -.392†  *n* = 25 |  |
| **ΔIL-6** |  |  | *r* = .013  *n* = 26 | *r* = .010  *n* = 25 | *r* = -.113  *n* = 25 |  |
| **ΔTNF-α** |  |  |  | *r* = .070  *n* = 25 | *r* = -.376†  *n* = 25 |  |
| **ΔStress** |  |  |  |  | *r* = -.532*  *n* = 25 |  |
| Note. NC = naturally cycling; HC = hormonal contraceptive; IL-1β = interleukin one beta; IL-6 = interleukin six; TNF-α = tumor necrosis factor alpha; ** *p* ≤ .001, * *p* ≤ .050 † *p* ≤ .064. | | | | | | |

In third generation HC users, changes in cortisol levels following stress were significantly positively correlated with changes in IL-1β and changes in TNF-α levels, and changes in IL-1β and changes in TNF-α were significantly positively correlated with each other as well. Additionally, changes in IL-1β and changes in TNF-α levels were both marginally significantly negatively correlated with changes in subjective positivity of mood, while changes in subjective stress levels were significantly negatively correlated with changes in subjective positivity of mood. Overall, when third generation HC users experienced a rise in cortisol levels in response to stress, this rise was accompanied by rises in both IL-1β levels and TNF-α levels, which were accompanied by a more negative mood. A more negative mood was also accompanied by increased appraisals of subjective stress.

**Supplemental Discussion**

As an exploratory aim, we investigated differences in stress responses between women using different generations of HCs. See Table S13 for information about differences between the currently available generations of HCs. Although insufficiently powered, initial exploratory results revealed that, compared to users of other HC generations, second generation users exhibited a more positive mood, and less subjective stress following the TSST. Additionally, compared to users of other HC generations, third generation HC users had a larger cortisol response to the stressor, heighted IL-1β levels that did not decrease in response to the stressor, and low baseline levels of TNF-α. In users of all three generations of HCs, rises in cortisol levels were accompanied by rises in TNF-α levels. In first generation HC users, rises in cortisol were also accompanied by rises in IL-6 levels, while in third generation HC users, rises in cortisol were instead accompanied by rises in IL-1β levels. These rises in levels of proinflammatory cytokines, for first and third generation HC users, were also accompanied by a more negative mood following the TSST.

| **Table S13**  *Generations of HC Progestins* | | | | | |
| --- | --- | --- | --- | --- | --- |
|  | **First**  **Generation** | | **Second Generation** | **Third Generation** | **Fourth Generation** |
| **Progestin Names** | | - Norethindrone/ Norethisterone acetate - Ethynodiol acetate - Medroxy-progesterone acetate | - Levonorgestrel - Norgestrel | - Desogestrel / Etonogestrel - Gestodene - Norgestimate | - Drospirenone - Dienogest |
| **HC Types** | | - Oral HC - Hormonal therapy - Depo-Provera (injectable) | - Oral HC - Hormonal IUD - Emergency contraception | - Oral HC - Nexplanon (implant) Nuva Ring (insertable) - Hormone therapy | - Oral HC - Hormonal therapy |
| **Androgenic Effects** | | - Variable in androgenicity – (low to moderate/highly androgenic) | - Highly androgenic | - Low androgenicity | - Anti-androgenic |
| **Progestational Effects** | | - Moderate/highly progestational | - Highly progestational | - Highly progestational | - Little progestational activity |
| Note: HC = hormonal contraceptive. (Dickerson & Bucci, 2002; Edwards, 2004) | | | | | |

Generally, second generation HC users exhibited less reactivity, both subjectively and biologically, to the TSST compared to users of the other generations of HCs, in contrast to results reported by Herrera and colleagues (2019), in which second generation HC users exhibited more reactivity to a stressor compared to first or third generation HC users. However, this reactivity in the previous work was driven by women in the inactive pill phase of their cycle, which might explain these disparate results. One potential explanation for the finding in the current work is that second generations HCs are most androgenic of the first three generations of HCs, which could imply that androgenicity of progestins in HCs influences women’s reactivity to stress. Another potential alternative or complementary explanation for this finding is that second generation HCs also typically contain the lowest doses of progestins, which could indicate that HC use impacts stress reactivity in a dose dependent fashion. However, as the sample size for this group was smaller than that of the other groups of HC users in the current study, these results should be interpreted with some caution.

Additionally, users of third generation HCs displayed a larger cortisol response to stress compared to users of other generations of HC users, and heightened IL-1β levels following stress. As third generation HCs are the least androgenic of the first three generations of HCs, these results, again, could suggest that differences in androgenicity are mechanistically responsible for differences in women’s responses to acute stress between users of different generations of HCs.

In general, the extant literature investigating differences between users of different types of HCs is limited to few studies conducted with small sample sizes, preventing researchers from investigating these differences appropriately. Large, well-controlled studies are needed, to allow researchers the power to investigate outcomes associated with different types of HC use, including differences based on mode of administration, dosage of synthetic estradiol, dosage of progestins, type of progestin, generation of progestin, androgenicity, progestinicity, and estrogenicity of progestins, alongside of individual differences in women, such as duration of HC use, age of HC use onset, endogenous sex steroid hormone levels, chronic stress exposure, and exposure to early-life stress, all of which likely interact to predict differential outcomes for women using HCs, in relation to their stress reactivity, their moods, and other potential unintended consequences associated with HC use (see Hill & Mengelkoch, 2022, for discussion).

References

Brynhildsen, J. (2014). Combined hormonal contraceptives: prescribing patterns,

compliance, and benefits versus risks. *Therapeutic Advances in Drug Safety*, *5*(5),

201-213. <https://doi.org/10.1177/2042098614548857>

Gelman, A., Hill, J., & Yajima, M. (2012). Why we (usually) don’t have to worry about multiple comparisons. *Journal of Research on Educational Effectiveness*, *5*(2), 189–211. <https://doi.org/10.1080/19345747.2011.618213>

Hall, K. S., & Trussell, J. (2012). Types of combined oral contraceptives used by US

women. *Contraception*, *86*(6), 659-665.

<https://doi.org/10.1016/j.contraception.2012.05.017>

Herrera, A. Y., Faude, S., Nielsen, S. E., Locke, M., & Mather, M. (2019). Effects of

hormonal contraceptive phase and progestin generation on stress-induced cortisol and

progesterone release. *Neurobiology of Stress*, *10*, 100151.

<https://doi.org/10.1016/j.ynstr.2019.100151>

Hill, S. E., & Mengelkoch, S. (2023). Moving beyond the mean: Promising research

pathways to support a precision medicine approach to hormonal

contraception. *Frontiers in neuroendocrinology*, *68*, 101042.

<https://doi.org/10.1016/j.yfrne.2022.101042>

Lawrence, J. D. (2019). Familywise and per‐family error rates of multiple comparison procedures. *Statistics in Medicine*. <https://doi.org/10.1002/sim.8190>

Perneger T. V. (1998). What's wrong with Bonferroni adjustments. *BMJ (Clinical research ed.)*, *316*(7139), 1236–1238. <https://doi.org/10.1136/bmj.316.7139.1236>

Rothman K. J. (1990). No adjustments are needed for multiple comparisons. *Epidemiology (Cambridge, Mass.)*, *1*(1), 43–46.

Rubin, M. A. (2017b). Do *p* Values Lose Their Meaning in Exploratory Analyses? It Depends How You Define the Familywise Error Rate. *Review of General Psychology*, *21*(3), 269–275. <https://doi.org/10.1037/gpr0000123>

Steegen, S., Tuerlinckx, F., Gelman, A., & Vanpaemel, W. (2016). Increasing Transparency Through a Multiverse Analysis. *Perspectives on Psychological Science*, *11*(5), 702–712. <https://doi.org/10.1177/1745691616658637>

Glickman, M. E., Rao, S. R., & Schultz, M. B. (2014). False discovery rate control is a recommended alternative to Bonferroni-type adjustments in health studies. *Journal of Clinical Epidemiology*, *67*(8), 850–857. <https://doi.org/10.1016/j.jclinepi.2014.03.012>

Moran. (2003). Arguments for rejecting the sequential Bonferroni in ecological studies.

Oikos, 100(2), 403–405. <https://doi.org/10.1034/j.1600-0706.2003.12010.x>

Peña, E. A., Habiger, J. D., & Wu, W. (2011). Power-enhanced multiple decision functions controlling family-wise error and false discovery rates. *Annals of statistics*, *39*(1), 556–583. <https://doi.org/10.1214/10-aos844>

Frane, A. V. (2016). Some clarifications regarding multiple comparisons. *Annals of Cardiac Anaesthesia*. <https://doi.org/10.4103/0971-9784.173033>

R Core Team (2021) R: A Language and Environment for Statistical Computing. R

Foundation for Statistical Computing, Vienna.
 <https://www.R-project.org>

Wilson D. J. (2019). The harmonic mean *p*-value for combining dependent

tests. *Proceedings of the National Academy of Sciences of the United States of*

*America*, *116*(4), 1195- 1200. <https://doi.org/10.1073/pnas.1814092116>

Wilson DJ (2019). harmonicmeanp: Harmonic Mean p-Values and Model Averaging by Mean Maximum Likelihood. R package version 3.0,  https://CRAN.R- project.org/package=harmonicmeanp.

1. Specifically, the largest differences in this study were observed between women taking second and third generation HCs, with those taking first generation HCs exhibiting stress responses more similar to those taking third generation HCs than second generation HCs. [↑](#footnote-ref-1)
